# Transition Metal Dichalcogenide Dimer Nanoantennas for Tailored Light–Matter Interactions

**DOI:** 10.1021/acsnano.2c00802

**Published:** 2022-04-06

**Authors:** Panaiot G. Zotev, Yue Wang, Luca Sortino, Toby Severs Millard, Nic Mullin, Donato Conteduca, Mostafa Shagar, Armando Genco, Jamie K. Hobbs, Thomas F. Krauss, Alexander I. Tartakovskii

**Affiliations:** † Department of Physics and Astronomy, University of Sheffield, Sheffield S3 7RH, U.K.; ‡ Department of Physics, University of York, York YO10 5DD, U.K.; § Chair in Hybrid Nanosystems, Nanoinstitute Munich, Faculty of Physics, Ludwig-Maximilians-Universität, München 80539, Munich, Germany

**Keywords:** nanophotonics, Mie resonators, transition metal dichalcogenides, photoluminescence enhancement, Purcell enhancement, optical trapping

## Abstract

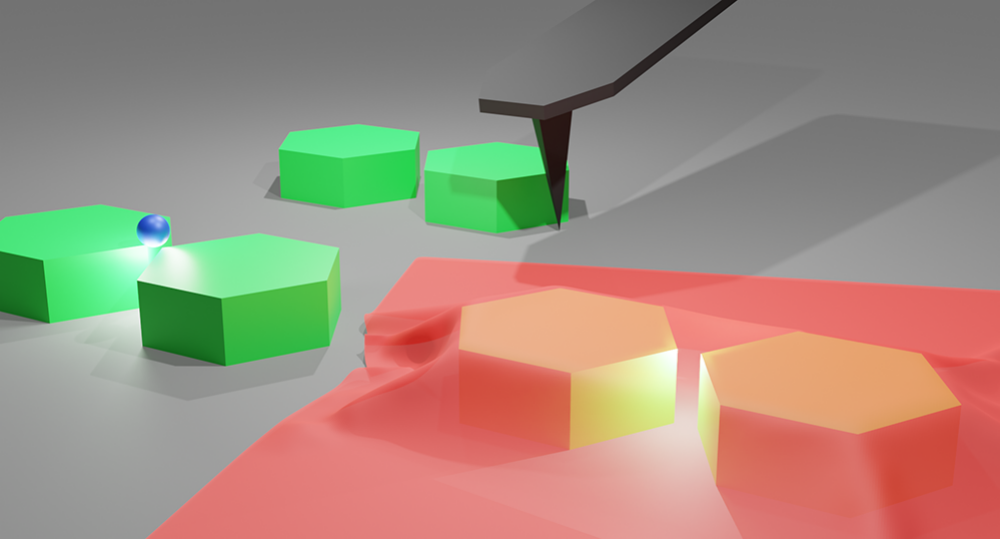

Transition metal dichalcogenides have emerged as promising materials for nanophotonic resonators because of their large refractive index, low absorption within a large portion of the visible spectrum, and compatibility with a wide range of substrates. Herein, we use these properties to fabricate WS_2_ double-pillar nanoantennas in a variety of geometries enabled by the anisotropy in the crystal structure. Using dark-field spectroscopy, we reveal multiple Mie resonances, to which we couple WSe_2_ monolayer photoluminescence and achieve Purcell enhancement and an increased fluorescence by factors up to 240 for dimer gaps of 150 nm. We introduce postfabrication atomic force microscope repositioning and rotation of dimer nanoantennas, achieving gaps as small as 10 ± 5 nm, which enables a host of potential applications, including strong Purcell enhancement of single-photon emitters and optical trapping, which we study in simulations. Our findings highlight the advantages of using transition metal dichalcogenides for nanophotonics by exploring applications enabled by their properties.

Transition metal dichalcogenides (TMDs) have drawn large scientific and technological interest in the past decade since the discovery of a direct band gap in monolayers due to quantum confinement effects,^1^ which in conjunction with reduced dielectric screening leads to strongly bound excitons.^2^ These layered materials found their way to research involving integration with nanophotonic structures such as plasmonic and dielectric cavities to realize both weak and strong coupling,^3−7^ low-threshold lasing,^8^ Purcell^9,10^ and quantum efficiency enhancement^11^ of single-photon emitters (SPEs), as well as coupling to collective resonances found in periodic structures.^12,13^ In these studies, the use of TMDs was limited to single- and few-layer samples focusing on coupling emitted light from 2D semiconductors to resonances and cavity modes in different material systems.^14^

Alternatively, the fabrication of a photonic resonator from a layered material similar to TMDs was first achieved in hexagonal boron nitride (hBN). Recent reports utilized electron beam induced and reactive ion etching of hBN to fabricate suspended one- and two-dimensional photonic crystal cavities as well as ring resonators, circular Bragg gratings, and waveguides.^15,16^ Moreover, hBN microcavity-like structures have been shown to control the spontaneous emission rate of excitons in MoS_2_ monolayers.^17^ Microrotator structures, twisted with an atomic force microscope (AFM) cantilever tip, have also been shown to facilitate the control of second harmonic generation (SHG) enhancement in hBN^18^ as well as the properties of electronic devices.^19^ Photonic resonators fabricated in TMDs, however, have only recently been reported even though these materials offer a number of advantages. The refractive index of WS_2_ in the visible range (*n* > 4)^20^ is higher than that of hBN (*n* ≈ 2.1)^21^ or other high-index dielectrics traditionally used to fabricate nanophotonic resonators such as gallium phosphide (*n* ≈ 3.5)^22^ or silicon (*n* ≈ 3.8).^22−25^ TMDs also often maintain a sizable transparency window in the visible^26^ range and offer advantages due to their layered nature, such as large optical anisotropy^27^ and adhesion to a large variety of substrates owing to their van der Waals attractive forces.^28^ These properties offer the possibility of producing a highly contrasting refractive index boundary by deposition of TMD crystals onto low refractive index materials such as SiO_2_,^29^ thereby providing a straightforward route to highly confined optical resonances.

Recent reports of TMD photonic structures have demonstrated strong coupling using WS_2_ photonic crystals,^30^ gratings,^31^ nanoantenna resonators,^20^ as well as TMD bulk flakes.^32^ Waveguiding or quasi-waveguiding has also been achieved in monolayer WS_2_ photonic crystals^33^ and bulk TMD flakes.^27^ TMD nanodisk Mie resonators, hosting nonradiative anapole modes, have also been fabricated to show second and third harmonic generation enhancement^34^ and Raman scattering enhancement.^35^ Numerical studies have explored the possibility of entire optical circuits in TMDs, including rib waveguides, photonic crystal cavities, and electro-optic modulators.^36^ Further theoretical reports enable the realization of MoS_2_ nanoresonator modes^37^ as well as WS_2_ nanoantenna metasurface resonances^38^ characterized as bound states in continuum.

Photonic structures with gaps smaller than 20 nm and a double-vertex geometry are highly desirable because of the possibility of strong confinement of electric and magnetic fields due to the boundary conditions on the normal and parallel components of the electric field at a sharp refractive index contrasting boundary.^39^ The high fields are a prerequisite for large radiative rate enhancements of emitters as evidenced from tip cavity structures in photonic crystal nanobeam cavities^39^ and plasmonic bowtie antennas.^40^ Incidentally, such large electric field intensities can also lead to stable optical trapping and therefore precise positioning of nanoparticles such as quantum dots or polystyrene beads, which closely resemble the size and refractive index of large proteins. This is due to an attractive Lorentz force in the direction of an electromagnetic hotspot under optical excitation, which is dependent on the particle size, refractive index, the input pump power, and the energy confinement provided by the photonic environment.^41^ Previous reports of nanoantenna optical trapping utilize plasmonic resonators;^42^ however, they suffer from large changes in temperature leading to loss in stability as well as quenching of emission due to increased optical absorption processes.^25,43^ Alternatively, dielectric nanoresonators, such as silicon dimer nanoantennas,^25,44^ can be advantageous for optical trapping in different applications, including biological nanoparticles at risk of degrading because of heating effects as well as quantum dot positioning without emission quenching. The large field confinement in closely spaced double-vertex structures, which is advantageous for Purcell enhancement as well as optical trapping, may be achieved in WS_2_ by using the etching anisotropy of the crystallographic axes^45^ and the weak van der Waals forces.

In this work, we pattern nanoantenna structures into thin WS_2_ crystals. We exfoliate 25–500 nm thick flakes of WS_2_ onto a SiO_2_ substrate and utilize established nanofabrication techniques such as electron beam lithography (EBL) and reactive ion etching (RIE) to define submicrometer nanoantennas with nanometer scale gaps. We observe that WS_2_ can be selectively fabricated in circular, square, or hexagonal geometries with potentially atomically sharp edges and vertices depending on the etching recipe used. Dark-field spectroscopy of single (monomer) and double (dimer) nanopillar resonators reveals geometric Mie resonances, which we compare with finite-difference time-domain (FDTD) simulations. We transfer a monolayer of WSe_2_ onto an array of fabricated dimer nanoantennas and observe photoluminescence enhancement factors of more than 240 on the structures when compared to emission from regions on flat SiO_2_. We also observe polarization-dependent PL emission aligned with the dimer axis and lifetime shortening by a factor of nearly 2, confirming the coupling of the WSe_2_ monolayer emission to the photonic resonances of the nanoantennas and yielding a Purcell factor lower bound of 1.85. Subsequently, we utilize contact mode atomic force microscopy (AFM) as a postfabrication step to reposition the constituent nanopillars of dimer nanoantennas achieving gaps of 10 ± 5 nm. We further numerically study the viability of utilizing dimer nanoantennas for the enhancement of single-photon emission rates. We simulate the electric field confinement as well as the Purcell factor for an SPE positioned at the hotspots of the dimer nanoantenna mode for gap sizes smaller than 20 nm, which we name ultrasmall. These simulations yield electric field intensity enhancements of >10^3^ compared to vacuum and Purcell factors of >150 for hexagonal and square geometries. We subsequently numerically explore the prospect of using WS_2_ dimer nanoantennas with ultrasmall gaps in optical trapping. For the smallest experimentally achieved dimer gap, we calculate attractive forces toward the electric field hotspots of >350 fN for colloidal quantum dots (QDs) and >70 fN for polystyrene beads (PBs) which closely emulate large proteins. Our experimental and numerical studies of TMD material photonic resonators explore different methods of radiative rate enhancement and optical trapping which may lead to a scalable route of fabricating Purcell enhanced SPEs for a variety of applications, including quantum computing and communication.

## Results and Discussion

### Fabrication of Nanoantennas

**Figure 1:**
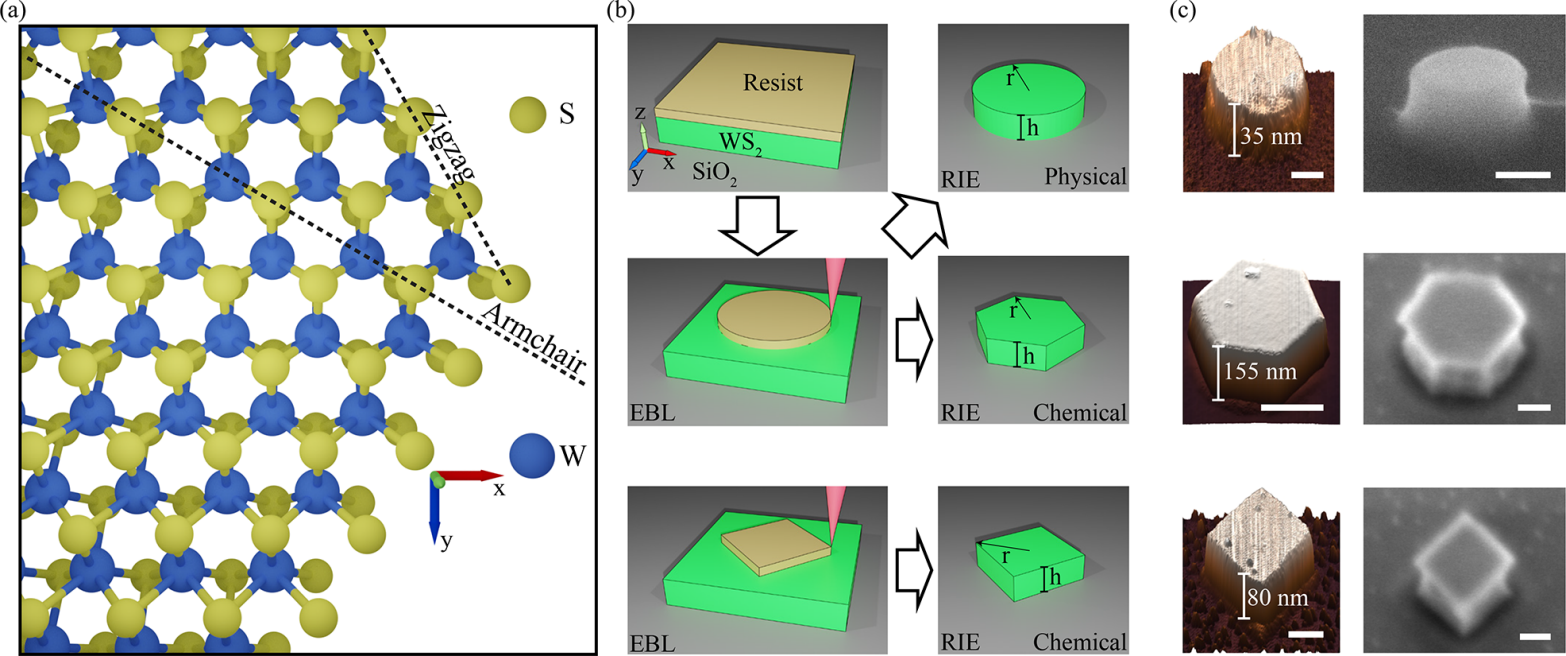
Fabrication procedure for WS_2_ nanoantennas. (a) Representation of a top view of a single layer of WS_2_ found at the top of a hexagonal nanoantenna at the position of the vertex. The zigzag axis defines the edges of the structure because of its higher stability as shown by the two bonds on outlying sulfur atoms when compared to the armchair axis with its single bond for outlying sulfur atoms. (b) Fabrication steps and their order following the black outline arrows. The first step includes spinning of resist onto a WS_2_ flake. The second step is patterning and development after electron beam lithography into a circular or square geometry. The final step is reactive ion etching using either a more physical or chemical etching recipe. The height (*h*) is defined by the thickness of the original flake. The radius (r) is defined as the distance from the center of the structure to an outside vertex for hexagonal and square geometries. (c) AFM and SEM images of fabricated nanoantennas. The left column shows 3D representations of AFM scans of circular, hexagonal, and square nanoantennas in the upper, middle, and lower row, respectively. Scale bars in AFM scans = 200 nm. The right column shows SEM images of similar structures taken at a 60° tilt of the sample. Scale bars in SEM images = 100 nm.

WS_2_ consists of covalently bonded monolayers (Figure 1a) with a hexagonal crystal structure held together by van der Waals forces in bulk crystals. We mechanically exfoliated WS_2_ flakes onto a 290 nm SiO_2_ on silicon substrate with thicknesses ranging from 25 to 500 nm. Figure 1b shows a schematic representation of the subsequent fabrication process. We spun a positive resist onto the sample and patterned circular disks and squares with varying radii using EBL. After development, we transferred the pattern into the WS_2_ crystals with RIE using two different recipes. Etching was terminated once the etch depth (estimated from the etching rate and time) matched the thickness of the exfoliated crystal, defining the nanoantenna height (see Methods). An anisotropic etch using a mixture of CHF_3_ and SF_6_ gases along with a high DC bias and low chamber pressure yielded circular nanopillars with vertical sidewalls, which resulted from the physical etching of the resist pattern into the WS_2_. Examples of completed structures are shown in the AFM and scanning electron microscopy (SEM) images in the upper row of Figure 1c.

Upon substitution of the CHF_3_ with additional SF_6_ gas, a reduction of the DC bias, and an increase in the chamber pressure, the resulting nanoantennas exhibited a hexagonal geometry with a radius defined from the center of the structure to one of the vertices at the edge, as shown in the middle right panel of Figure 1b. This definition corresponds to the radius of the previously circular resist pattern. The physical etching mechanism was suppressed, and the increased proportion of reactive fluorine radicals ensured a dominant chemical etch, which preferentially removed the WS_2_ crystal in the armchair crystal axis leading to zigzag terminated sidewalls at 120° angles to each other following the crystal symmetry. This agrees with DFT results, which also predict that zigzag edges are more stable.^46,47^ The AFM and SEM images in the middle row of Figure 1c display an example of hexagonal nanoantennas.

The final geometry we achieved was that of a square, which resulted from a combination of resist patterning and chemical etching of the WS_2_. We defined a square resist pattern with sides oriented parallel to the zigzag axis of the crystal. The subsequent chemical etching similarly removed the WS_2_ in the armchair crystal axes, ultimately leading to 90° angles describing a square shaped nanoantenna, examples of which are displayed in the lower panels of Figure 1c. The hexagonal geometry and, in part, the square nanoantenna geometry are formed because of the relative stability of the zigzag axis and can therefore lead to atomically sharp vertices.

### Photonic Resonances of WS_2_ Nanoantennas

We studied the fabricated structures using dark-field spectroscopy and compared the experimental results to FDTD simulations, which yielded close agreement. We identified an electric dipole resonance with small contributions from higher-order modes as well as anapole and higher-order anapole modes (see Supporting Information 1). As expected from Mie theory, the resonances forming in the studied nanoantennas red-shifted with increasing radius and blue-shifted with decreasing height. The ability to change the geometry of nanoantennas through the choice of etching recipe provides an additional, more precise tuning mechanism for the observed resonances (see Supporting Information 2).

**Figure 2:**
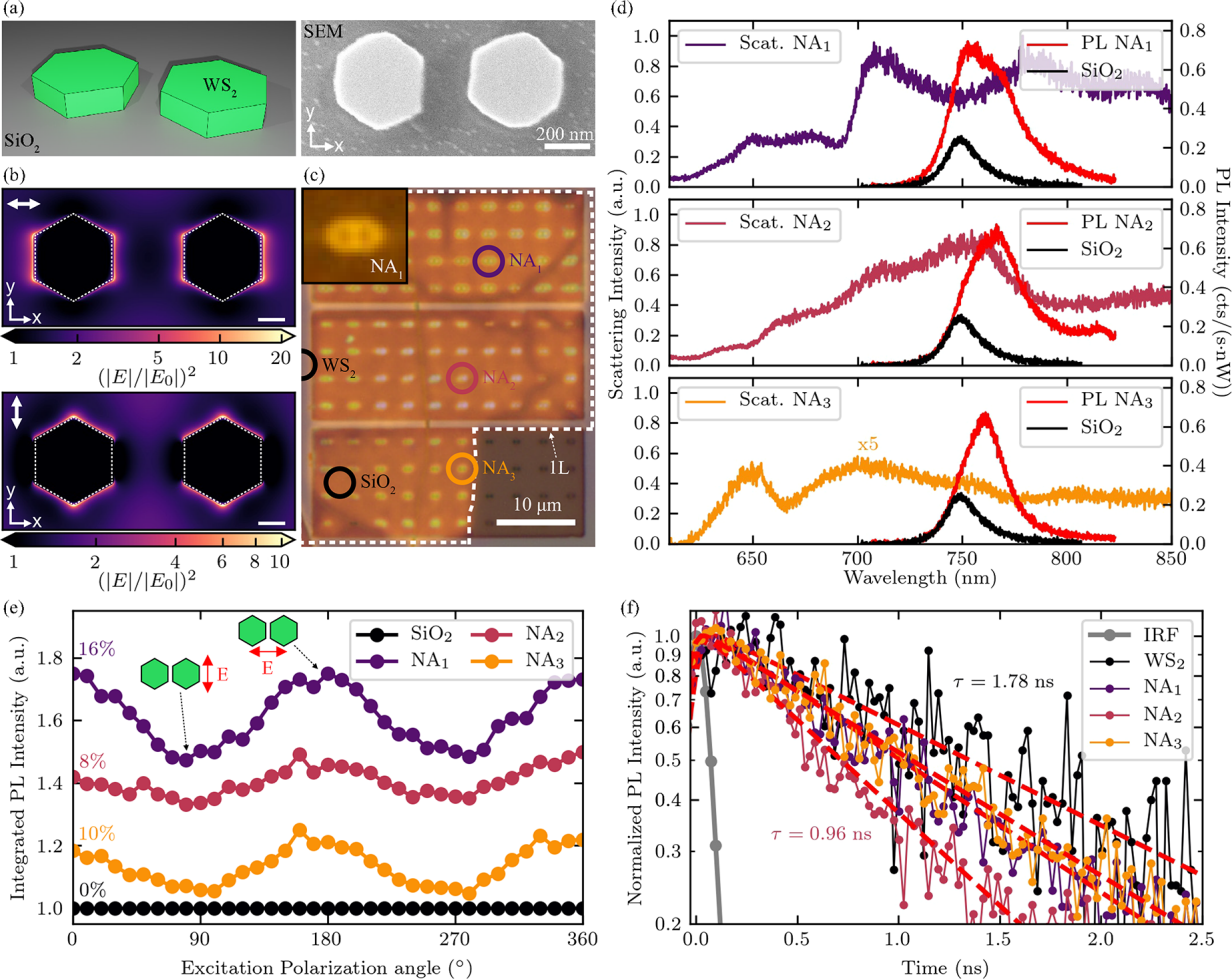
PL emission enhancement from monolayer WSe_2_ on WS_2_ hexagonal dimer nanoantennas. (a) Left panel: Schematic illustration of a hexagonal dimer nanoantenna. Right panel: SEM image of the top view of a fabricated hexagonal dimer nanoantenna. (b) Top view spatial distributions of the simulated electric field intensity 0.5 nm from the top surface of a WS_2_ dimer nanoantenna with a radius of 165 nm, a gap of 200 nm, and a height of 135 nm. Incident plane wave polarization is parallel to the double white arrow in the upper left corner of each panel. Hotspots of high electric field intensity form on the edges toward the gap and away from it in the upper panel (X-pol mode). Hotspots form at upper and lower edges of the nanostructure in the lower panel (Y-pol mode), yielding less confinement for this plane wave polarization. Axes (*x*, *y*) indicate cross-sectional surface similar to that in the right panel of a. Dashed white outlines represent the physical edges of the structures. Scale bars = 100 nm. (c) Photoluminescence microscope image overlaid on a bright field microscope image showing a map of the studied sample including the monolayer region. Bright emission is observed at nanoantenna sites. Inset: Magnified PL image of NA_1_ showing that bright emission regions follow the outline of the nanoantenna geometry. (d) Dark-field scattering spectra for three exemplary nanoantennas shown with colored circles in panel c. Photoluminescence spectra from the WSe_2_ monolayer on the nanoantenna sites (red) as well as from a flat region on SiO_2_ (black) are displayed as well. The PL intensity on all nanoantenna sites is enhanced compared to that from flat SiO_2_. The overlap between the scattering resonances and the monolayer PL spectrum from the nanoantenna sites suggests enhanced emission. (e) Excitation polarization-dependent integrated PL measured at the three nanoantenna sites. Degrees of linear polarization are shown on the left side of each curve. All nanoantennas exhibit a polarization dependence, which is aligned to the dimer axis suggesting coupling between the monolayer emission and the nanoantenna resonances. The intensity is normalized to that from a flat monolayer on SiO_2_ and vertically offset for illustration purposes. (f) Photoluminescence decay measured from the monolayer at each nanoantenna site and on bulk WS_2_ crystal. The measured lifetimes are as follows: WS_2_, 1.78 ± 0.08 ns; NA_1_, 1.34 ± 0.02 ns; NA_2_, 0.96 ± 0.01 ns; NA_3_, 1.45 ± 0.02 ns. Reduced lifetimes on nanoantennas suggests Purcell enhancement of emission.

Next we considered more complex architectures by placing a pair of hexagonal nanopillars in close proximity to form a dimer nanoantenna, as shown schematically in the left panel of Figure 2a. An SEM image of a fabricated structure is also displayed in the right panel of Figure 2a. Dark-field spectra of dimer nanoantennas also exhibit scattering Mie resonances as well as anapole modes which red-shift with increasing size (see Supporting Information 3). However, when two single nanopillars are placed in close proximity to form a dimer, their photonic resonances hybridize forming two cross-polarized modes with an energy splitting (see Supporting Information 4). One of these modes is excited with a polarization parallel to the axis connecting the midpoints of the single nanopillars (dimer axis), which we name the X-pol mode, while the other is excited perpendicularly, here named the Y-pol mode. An intriguing result from this hybridization is the increased electric field intensity surrounding the side-walls of the nanoantennas. For the X-polarized mode, high electric field intensity hotspots form in the gap separating the two nanopillars, as shown in the upper panel of Figure 2b. For the Y-polarized mode, these hotspots form at the upper and lower wall of each nanopillar, as shown in the lower panel of Figure 2b. This confinement of the electric field intensity is expected to induce a large density of optical states, suggesting that these nanoantennas are advantageous for Purcell enhancement of emission.

### WSe_2_ Monolayer Photoluminescence Enhancement

In order to investigate whether these nanostructures can be used for such an application, we transferred a monolayer of WSe_2_ onto an array of WS_2_ dimer nanoantennas with a varying radius and gap distance using an all-dry technique (see Methods). A photoluminescence (PL) image of the completed sample is shown in Figure 2c. The brighter emission surrounding the nanoantenna sites is a first indication of the enhanced PL emitted by the monolayer due to an interaction with the dimer nanoantennas. The shape of this bright emission follows the outer walls of the nanoantennas, as seen in the inset of Figure 2c, similar to the calculated higher electric field intensity regions shown in Figure 2b.

In order to experimentally evaluate the photonic response of the structures, we subsequently measured the dark-field spectra of three nanoantennas with a monolayer of WSe_2_ on top. These are displayed in Figure 2d together with PL emission from the monolayer measured at each nanoantenna site. These structures have a height of 135 nm, a gap of 150 nm, as well as a range of radii (NA_1_, *r* = 235 nm; NA_2_, *r* = 185 nm; NA_3_, *r* = 120 nm), representing an exemplary set of the measured nanoantennas from our sample, which included 86 dimers with nominal geometries corresponding to one of the three presented here. The overlap between the scattering Mie resonances and the PL spectrum suggests that all of the nanostructures may induce some emission enhancement; however, the strongest effect is expected from dimer NA_2_.

We subsequently carried out detailed room-temperature photoluminescence measurements in a micro-PL setup in order to study the enhanced emission from the WSe_2_ monolayer in more detail. The excitation source, a pulsed laser (80 MHz) at 638 nm, was chosen to be below the WS_2_ absorption edge so that it would be absorbed only in the WSe_2_ monolayer and not in the nanoantennas. The spectra recorded at the position of the three dimer nanoantennas are displayed in Figure 2d (red). These are compared to a PL spectrum measured from a flat portion of monolayer on SiO_2_ shown in black. The red-shift of the PL spectrum observed here is due to strain in the monolayer as it conforms to the nanoantenna geometry, which however provides only a negligible contribution to the large photoluminescence enhancement observed here as evidenced by a lack of brightening at the edges of the unpatterned bulk WS_2_ crystal regions in Figure 2c where the WSe_2_ monolayer is also strained.^48^ This suggests that the WS_2_ dimer nanoantenna platform is ideal to study strain effects in monolayer TMDs similar to dimers fabricated from other dielectrics.^11,48^ The luminescence intensity from the monolayer at the nanoantenna positions is 3.5–4 times brighter. As the excitation spot is much larger than the nanostructures, we defined an experimental enhancement factor ⟨EF⟩, similar to that in ref (7), in order to estimate the enhanced PL intensity:
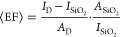
1where *I*_D_ and  are the spectrally integrated PL intensity measured on each dimer nanoantenna and on the flat SiO_2_ substrate, respectively. The area of each dimer nanoantenna and the area of the laser spot are represented as *A*_D_ and  respectively. Using this definition, we calculated enhancement factors of 120, 235, and 241 for nanoantennas NA_1_, NA_2_, and NA_3_ respectively, when compared to a flat monolayer on the SiO_2_ substrate. If monolayer emission from positions on bulk WS_2_ (seen at the left edge of Figure 2c) rather than on flat SiO_2_ is used for comparison, the enhancement factors are estimated to be 246, 486, and 533 for nanoantennas NA_1_, NA_2_ and NA_3_, respectively. This is due to the lower monolayer PL on bulk WS_2_ when compared to flat SiO_2_, which we largely attribute to charge transfer between the WSe_2_ and WS_2_ crystal.^49^ We also compared the experimental ⟨EF⟩ to simulations of the maximum fluorescence intensity enhancement in the vicinity of dimer nanoantennas with the same geometry and found order of magnitude agreement (see Supporting Information 5).

Another method of probing the photonic enhancement of the WSe_2_ emission due to the nanoantennas is to induce a linear polarization in the excitation source and rotate this with respect to the dimer axis. As the electric field intensity surrounding the nanoantennas is higher for the X-pol mode when compared to the Y-pol mode, which can be observed in Figure 2b, the PL intensity is also expected to increase for this polarization. In this experiment, the integrated intensity at each polarization angle was normalized to that from a monolayer region on flat SiO_2_, defined as *I*_D_(θ)/*I*_SiO_2__(θ), so as to highlight the effect of the nanoantenna photonic resonances. The results are shown in Figure 2e where all structures yield a degree of linear polarization of the photoluminescence, shown on the left of each plot, confirming the coupling of the emission to the nanoantenna resonances. Further evidence of photonic enhancement of the WSe_2_ monolayer due to an enhanced electric field intensity can be extracted from a comparison of absorption spectra measured at the position of the nanoantennas as well as on the flat SiO_2_ substrate (see Supporting Information 6).

The last experiment performed in order to study the photonic enhancement of WSe_2_ monolayer emission due to the WS_2_ dimer nanoantennas was a measurement of the PL decay time, displayed in Figure 2f. For this study, an avalanche photodiode was used as a detector with an instrument response function (IRF, gray) defined by the laser pulse (∼90 ps). A very low excitation power density of 0.02 μJ/cm^2^ was used in order to avoid exciton–exciton annihilation processes, which dominate in these materials at higher powers.^50^ The results yield single exponential decay lifetimes, which consist of contributions from both radiative and nonradiative recombination rates. An increase in the radiative rate due to photonic enhancement will lead to a decrease in the emission lifetime as this component is shortened. The intrinsic WSe_2_ monolayer PL lifetime at room temperature measured from the monolayer on the WS_2_ bulk crystal was τ = 1.78 ± 0.08 ns. The PL decay times measured for NA_1_, NA_2_, and NA_3_ are τ_1_ = 1.34 ± 0.02 ns, τ_2_ = 0.96 ± 0.02 ns, and τ_3_ = 1.45 ± 0.02 ns, respectively. These are lower than that measured on the bulk crystal, suggesting the presence of Purcell enhancement. If we assume that the nonradiative rate contribution to the lifetime is low, we can extract Purcell factors of 1.33, 1.85, and 1.23 for NA_1_, NA_2_, and NA_3_, respectively. However, previous measurements of the quantum efficiency of WSe_2_ monolayers report values ranging from 0.06% to 5%;^51−53^ therefore, the nonradiative recombination rate is much higher than the radiative. As the Purcell factor affects only the radiative component of the lifetime, the values extracted above are only lower bounds on the spontaneous emission enhancement factor, which may be much higher. The upper bound of the Purcell factor can be extracted from FDTD simulations, which yield a factor of nearly 40 for the geometry of NA_2_ (see Supporting Information 5). As suggested by the higher overlap of the PL emission with the scattering resonances of the nanoantenna, the shortest PL lifetime was measured for NA_2_ where the highest Purcell enhancement is expected. This suggests that the enhancement factor can be modulated by tuning the dimer nanoantenna resonances closer or further from the PL emission energy of the monolayer (see Supporting Information 5). We also varied the linear polarization of the excitation source and measured the PL decay time for the X-pol and Y-pol modes. For the majority of the measured nanoantenna sites, the X-pol mode yielded a lower lifetime than the Y-pol mode (see Supporting Information 7) as expected from the higher simulated electric field intensities, in Figure 2b, and therefore higher Purcell factors.

As further evidence of the photonic capabilities of WS_2_ dimer nanoantennas, we performed second harmonic generation enhancement experiments using an anapole resonance present in 60 nm high nanoantennas with a radius of 205 nm and a gap of 130 nm. The anapole resonance at a wavelength of 800 nm led to confinement of the excitation laser and a 7.2 times enhanced SHG signal when compared with bulk crystal. The enhancement also proved to be polarization-dependent in dimer nanoantennas, as opposed to monomers, as the electric fields are confined outside (inside) the nanostructure geometry for the X-pol (Y-pol) anapole mode. This behavior was confirmed by simulations of the confined electric energy, which also reveal that the SHG enhancement polarization orientation can be rotated with a change in excitation wavelength and its degree of linear polarization can be modulated with an increase in dimer gap (see Supporting Information 8).

### AFM Repositioning

The fabrication procedure outlined in Figure 1 yields minimum dimer separation gaps of 50 nm, which result in resonances with limited electric field intensity and therefore Purcell factors. We attempted to improve upon this by using the properties of layered materials to our advantage. The relatively weak van der Waals forces, which facilitate mechanical exfoliation of thin WS_2_ crystals, also allow for a weak adhesion of the fabricated structures to the SiO_2_/Si substrate. As a postfabrication procedure, we employed an AFM cantilever in order to translate one nanopillar with respect to the other and achieve gaps as small as 10 nm without damaging the nanoantenna. Smaller separation distances may be achievable; however, the accurate measurement of the gap width is a considerable challenge because of the finite size of the cantilever tip, which ranges from 1 to 10 nm.

**Figure 3:**
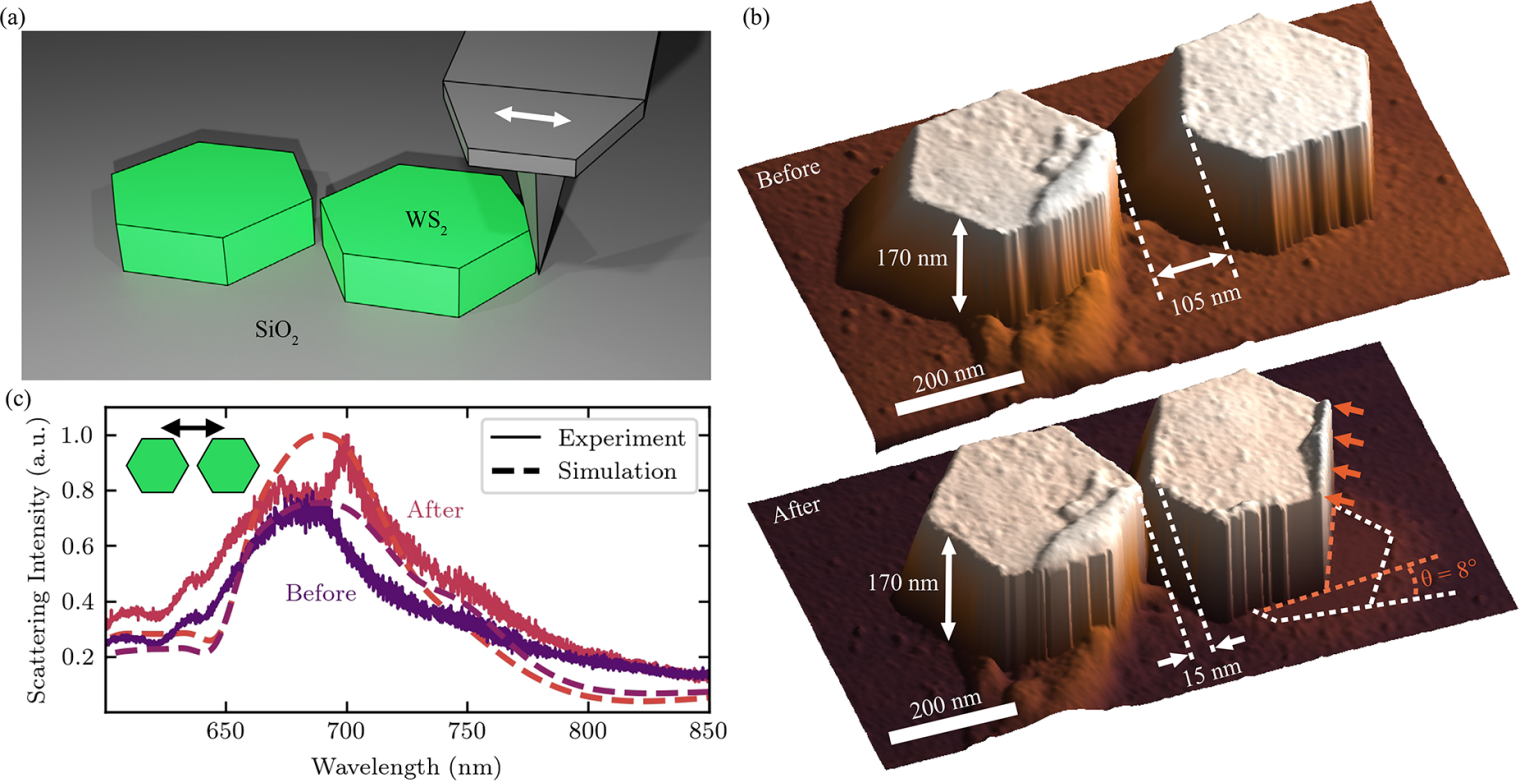
AFM repositioning of dimer nanoantennas. (a) Schematic illustration of the scanning technique used in contact mode AFM to translate and rotate single nanopillars of the dimer nanoantenna. (b) AFM scan of a dimer structure (*r* = 140 nm, *h* = 170 nm, gap = 105 nm) before and after manipulation. The dimer gap is reduced to 15 nm, and one pillar is rotated by 8° in order to position inside vertices closer to each other. (c) Dark-field scattering spectra of the dimer-nanoantenna for excitation parallel to the dimer axis (as shown in the inset) before and after repositioning compared to a simulated scattering cross section with gaps of 105 and 15 nm.

Nanoantennas were repositioned by using AFM in contact mode. Scanning parallel to the dimer axis in a small area immediately adjacent to one nanopillar with the scan slightly overlapping the distal edge of the nanoantenna forced the cantilever tip to displace the structure closer to the other. A schematic representation of the translation is shown in Figure 3a. Very fine positioning can be achieved using this method as shown in Figure 3b, where we translated one nanopillar with respect to the other, reducing the dimer gap separation from 105 nm to 15 ± 5 nm.

An additional advantage of using this repositioning method is the ability to rotate the nanopillars with great precision (<1°). As shown in the lower panel of Figure 3b, by scanning the AFM cantilever along a tangent of one of the nanopillars and contacting with only the edge, it was rotated by over 8°. Scanning closer to the midpoint of the nanopillar translated it closer to the other. These methods of contacting the AFM tip with the nanopillars allow for fine positioning of the inside vertices of the dimer. The repositioning procedure is iterative and entirely reversible, allowing alignment to be fine-tuned or completely changed as necessary. Further AFM scans of repositioned nanoantennas are displayed in Supporting Information 9, confirming the reproducibility of the procedure and showing the minimum gap separation we have achieved (10 ± 5 nm).

To confirm that the AFM repositioning modified the photonic response, we performed dark-field spectroscopy before and after the translation procedure. As shown in Figure 3c, the dipole resonance seen in scattering increased after repositioning for an excitation parallel to the dimer axis, which is the most sensitive configuration to changes in gap separation. The dashed curves in the same figure represent simulations of the geometry before and after repositioning with close agreement to experiment supporting the achievement of a 15 nm gap.

### Electric Field and Purcell Enhancement of Emission

Ultrasmall gaps, such as those achieved with the AFM repositioning technique, are expected to provide very high electric field confinements.^39^ We therefore simulated the electric field intensity and Purcell enhancement induced by three dimer designs, corresponding to the different geometries shown in Figure 1, with a gap of 10 nm. Each design was optimized for electric field confinement at the top surface of the dimer.

**Figure 4:**
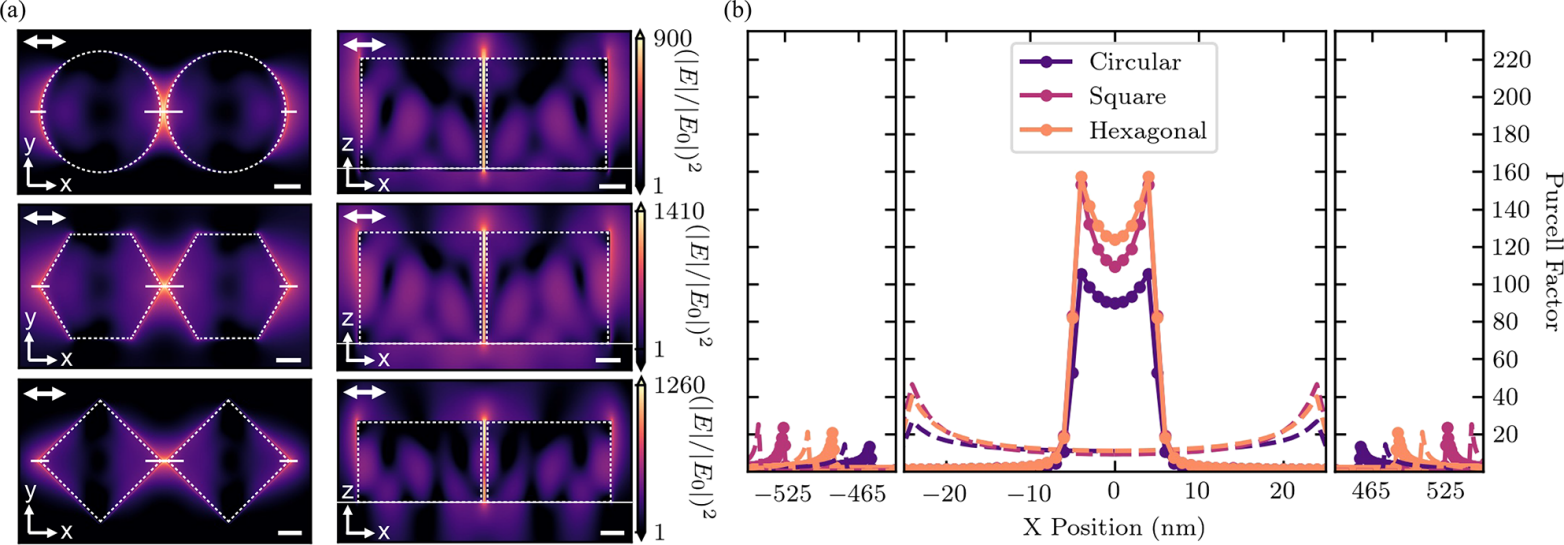
Simulations of the electric field hotspots and Purcell enhancement for single-photon emission. (a) Top-view and side-view spatial distributions of the electric field intensity in and surrounding optimized designs of each geometry of WS_2_ dimer nanoantenna at 10 nm separation gap. Circular design: *r* = 225 nm, *h* = 200 nm, wavelength = 751.5 nm. Hexagonal design: *r* = 240 nm, *h* = 200 nm, radius of curvature of vertices = 22 nm, wavelength = 749.5 nm. Square design: *r* = 260 nm, *h* = 150 nm, radius of curvature of vertices = 10 nm, wavelength = 697.5 nm. Hotspots of electric field confinement form at inner and outer edges of the dimer. Incident plane wave polarization (X-pol) is parallel to the double white arrow in the upper left corner of the panels. Axes indicate cross section with (*x*, *y*) indicating a surface 0.5 nm above the top of the dimer and (*x*, *z*) indicating a vertical cut through the dimer axis. Dashed white outlines represent the physical edges of the structures. Scale bars = 100 nm. (b) Purcell enhancement for a dipole placed at different positions with polarization parallel to the dimer axis, 0.5 nm above the top surface of the structures. Solid dots and curves indicate dimers with a gap of 10 nm, and dashed curves indicate dimers with a gap of 50 nm. Placement positions of the dipole above the dimer are shown as solid white lines in panel a.

The electric field intensity spatial distribution is shown 0.5 nm above the top surface of the dimers in the left column of Figure 4a and as a cross-sectional cut along the *z*-axis through the middle of the individual nanopillars in the right column of Figure 4a. Each distribution was calculated at the wavelength of the maximum electric field intensity (751.5, 749.5, and 697.5 nm for the circular, hexagonal, and square geometries, respectively). The radii of the optimized geometries are *r* = 225 nm, *r* = 240 nm, and *r* = 260 nm for the circular, hexagonal, and square geometries, respectively, while their heights are *h* = 200 nm, *h* = 200 nm, and *h* = 150 nm. In order to closely approximate realistic structures, which can be fabricated, we measured the radius of curvature of the vertices of fabricated hexagonal and square dimer nanoantennas using atomic force microscopy. This yielded vertex radii of curvature as low as 22 nm for the hexagonal and 10 nm for the square geometry (see Supporting Information 10). These values were then subsequently taken into account in the simulation of the hexagonal and square geometries. The electric field hotspots forming at the vertices between the two nanopillars for an incident plane wave polarized parallel to the dimer axis exhibit intensities of more than 10^3^ compared to vacuum as shown in Figure 4a. We compare the hotspots simulated for the three geometries achievable through the fabrication process. The hexagonal and square shaped nanoantennas induce a higher electric field intensity confinement than the circular design, with the hexagonal geometry inducing the largest enhancement. Smaller vertex radii of curvature for the latter two geometries, which may be achievable with the chemical etching recipe discussed earlier, are expected to yield even higher electric field intensity enhancements (see Supporting Information 10).

Furthermore, we evaluated the Purcell factor for a single-photon source positioned onto these structures by simulating a dipole emitter at positions along the dimer axis with a polarization parallel to the same axis. It is displaced by 0.5 nm from the top surface of the structure, and its emission wavelength was set to the one used for the respective geometry in Figure 4a. The calculated Purcell factors at each position are shown in Figure 4b. Solid white lines in the left panels of Figure 4a indicate the simulated positions of the emitter. The maxima seen at positions corresponding to the inner edges of the nanoantenna yield the largest results (>100) as expected from the electric field hotspots seen in Figure 4a. Weakly confined hotspots at the outside edges of the structure also exhibit local maxima in the Purcell factor with values as high as 20. For the minimum achieved gap separation in the postfabrication AFM repositioning, the hexagonal geometry exhibits the highest Purcell factor within its hotspot (157) with the square as a close second (153). The circularly shaped dimer leads to a maximal Purcell factor of 105. We have also simulated the Purcell factors for the minimum gap separation achievable without the use of AFM repositioning (50 nm), which are shown in Figure 4b as dashed curves. This yields much smaller Purcell enhancements (as high as 46). There are several degrees of freedom in the fabrication process that can be used to modulate both the electric field intensity and the Purcell enhancements expected for these dimer structures. One method is to vary the gap separation of the dimer, leading to an exponential decrease in the electric field intensity and Purcell factor by 1 order of magnitude for a gap of 100 nm when compared to a gap of 10 nm. A similar decrease in both factors can also be achieved by rotation of one nanopillar with respect to the other (see Supporting Information 11). Such modulations can be experimentally achieved through the use of nanofabrication techniques or AFM repositioning.

### Dimer Nanoantenna Optical Trapping

**Figure 5:**
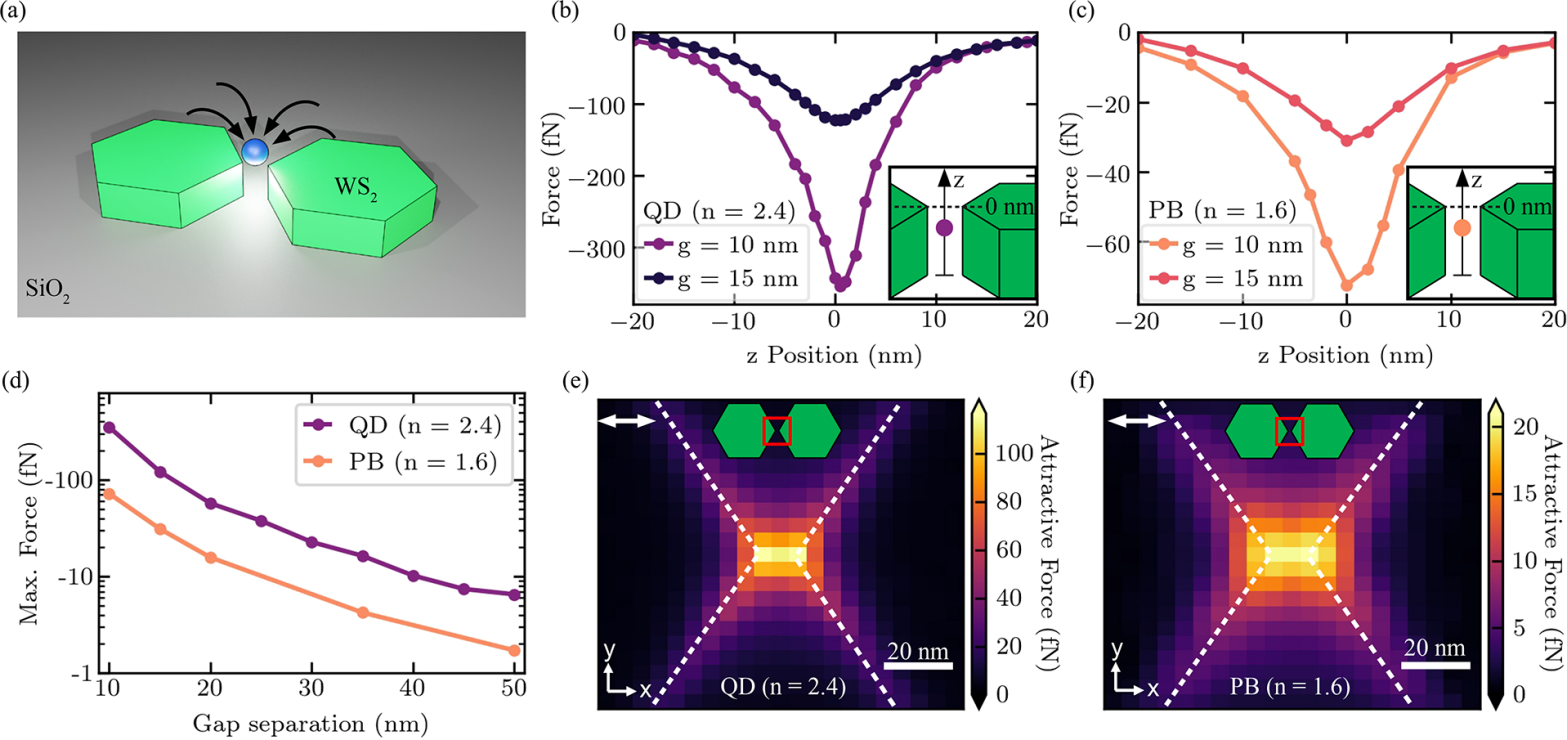
Optical trapping simulations of nanoparticles using WS_2_ hexagonal nanoantennas. (a) Schematic illustration of optical trapping of a nanoparticle in water through the use of WS_2_ hexagonal dimer nanoantenna hotspots. (b and c) Comparison of the optical force for a spherical nanoparticle (*r* = 5 nm) placed in the middle of the dimer nanoantenna (*r* = 240 nm, *h* = 200 nm) gap, along the *z*-axis for gaps of 10 and 15 nm. The trapping force is calculated at the wavelength of its maximum value for a QD (b) (763 nm) and a PB (c) (755 nm). Insets illustrate the positioning of the respective nanoparticle with the zero position at the top surface of the nanoantenna. (d) Maximum optical force applied to a QD (purple) and a PB (yellow) at the top surface of a dimer nanoantenna (*r* = 240 nm, *h* = 200 nm) with a varying gap separation. (e and f) Attractive force distribution for a nanoparticle in a plane 5 nm from the top surface of a dimer nanoantenna (*r* = 240 nm, *h* = 200 nm, *g* = 10 nm) centered at the gap. The trapping force is calculated at the wavelength of its maximum value for a QD (e) (763 nm) and a PB (f) (755 nm). The double white arrow in the upper left portion of the figure identifies the pump polarization along the dimer axis (X-pol). Dashed white lines identify the physical edges of the structure. Inset schematics highlight the position of the plane over which the optical force is simulated. Maximum force values coincide with the electric field hotspots. Input pump power density used for all simulations is 10 mW/μm^2^.

In order to study the potential of ultrasmall gap WS_2_ dimer nanoantennas for optical trapping, we perform numerical simulations based on the finite element method (FEM) to determine the Maxwell stress tensor (MST) and calculate the force exerted on small dielectric particles. The simulation geometry consists of the earlier optimized hexagonal nanoantenna with the addition of a nanosphere (*r* = 5 nm) with a refractive index corresponding to either an approximated colloidal quantum dot (*n* = 2.4)^44^ or a polystyrene bead (*n* = 1.6), which closely mimics the refractive index and size of a large protein.^54^ Because optical trapping experiments often require a suspension of the nanoparticles in a solution, we set the background refractive index to that of water. For all simulations, we use an experimentally feasible pump power density of 10 mW/μm^2^. A schematic representation of the simulation is shown in Figure 5a.

We explore the optical force applied to the QD and the PB, shown in panels b and c of Figure 5, respectively, as they are translated along the *z*-axis in the middle of the dimer gap. We simulate the forces for two gap separations of the dimer hexagonal nanoantenna (10 and 15 nm) and observe an attractive (negative) force, which is maximized at the top surface of the nanoantenna where the electric field hotspots are formed. We observe a maximum optical force of 353 fN on the QD and 73 fN on the PB for the dimer nanoantennas with a 10 nm separation. For the larger dimer gap (15 nm), this reduces to 123 fN on the QD and 31 fN on the PB. We also study the dependence of this maximum optical force in the electric field hotspots on the dimer gap, which is varied from 10 to 50 nm. As shown in Figure 5d, the maximum attractive force imparted on the nanoparticles decreases exponentially by 1 order of magnitude as expected from the decay of the electric field intensity with an increased separation (see Supporting Information 11).

We subsequently explore the lateral spatial dependence of the optical force by simulating the QD and PB in a plane, which is 5 nm above the top surface of the dimer nanoantenna with a 10 nm gap, as shown in Figure 5e,f. This is an emulation of the position of nanoparticles placed onto the top surface of the dimer. The optical force, as expected, is maximized at the position of the electric field hotspots yet still maintains values of more than 100 fN for the QD and 20 fN for the PB.

## Conclusion

We have fabricated and characterized both monomer and dimer WS_2_ nanoantennas with the aim to highlight the advantages of their use in photonic applications such as tunable photoluminescence enhancement, polarization-dependent SHG enhancement, SPE enhancement, and optical trapping, thereby broadening the versatility of TMD nanoantennas. Dark-field studies on monomer and dimer nanoantennas reveal a straightforward approach for tuning resonances in the structures in order to fit with different applications by varying the radius, height, or geometry. For the first time, we couple monolayer WSe_2_ emission to dimer resonances in the same, TMD, material system and achieve PL enhancement factors of more than 240 and a Purcell enhancement factor lower bound of nearly 2 for a large dimer gap of 150 nm. Through the use of a dimer anapole mode we show polarization-dependent SHG enhancement, not present in bulk TMD flakes or monomer nanoantennas, that can be rotated by a change in excitation polarization which may prove advantageous for the realization of optical logic gates.^55^ Post-fabrication repositioning of dimer nanoantennas utilizing AFM led us to attain ultrasmall gaps of 10 ± 5 nm, on the order of the feature resolution limit set by FIB milling,^56^ which we achieve through a more precise, less damaging technique. Although a previous report has demonstrated AFM positioning of plasmonic bowtie nanoantennas,^57^ this technique has only become possible for dielectric nanoantennas made from layered materials because of their intrinsic van der Waals attractive forces. This allows us to achieve the smallest separation recorded for a dielectric resonator defined through the use of electron beam lithography followed by reactive ion etching.^25,58^ Another advantage of this method is the possibility to change the relative orientation of individual nanopillars, thereby aligning their potentially atomically sharp vertices in closer or further proximity.

The numerical simulation studies of dimer nanoantennas exhibiting an ultrasmall gap yield highly confined electric field intensity hotspots (10^3^ enhancement compared to vacuum). We predict the practical utility of WS_2_ nanoantennas for radiative rate enhancement of single-photon emission with Purcell factors of up to 157 for a hexagonal and 153 for a square geometry. The expected quantum emission enhancement in the dimer nanoantennas is higher than the largest currently achieved in photonic crystal cavities^59^ because of the previously inaccessibly small proximity attained with the AFM repositioning method. We also explore two different routes to modulate the photonic enhancement through variation of the dimer gap as well as the relative rotation of the individual nanopillars, which are both controllable either through the fabrication process or, to a greater precision, the postfabrication repositioning. These methods of controlling the emission properties of single-photon sources may prove useful for WSe_2_ SPEs, which form at high strain gradients in monolayers transferred onto dimer nanoantennas collocated with the electric field hotspots.^48^ Previous reports have shown quantum efficiency enhancement for WSe_2_ SPEs^11^ as well as rotation of the emitter dipole moment due to a change in the strain gradient,^60^ both of which can be controlled by a change in separation and rotation of individual nanopillars in the dimer nanoantenna through AFM repositioning.

We further explored a route to the precise positioning of SPEs by simulating optical trapping forces due to the highly confined electric field in the gap of the dimer nanoantennas. We calculated a maximum attractive force of 353 fN for a colloidal quantum dot and 73 fN for a protein-like, polystyrene bead both with a radius of 5 nm under a pump power density of 10 mW/μm^2^. When compared to previous examples of dielectric nanoantennas for optical trapping, the WS_2_ dimers yield higher attractive forces by a factor of >83 for QDs^44^ and >40 for PBs^25^ with the same size and under the same pump power conditions. Therefore, WS_2_ dimer nanoantennas with ultrasmall gaps show great potential for applications of stable trapping of very small nanoparticles with a moderate optical power. This once again highlights the advantage of the AFM repositioning technique to reduce the dimer gap below the limits available to standard nanofabrication. The large Purcell enhancements and optical trapping forces predicted for WS_2_ dimer nanoantennas with ultrasmall gaps highlight the potential for TMD nanoresonator research and applications. This is possible because of the refractive index and van der Waals forces, which allow the formation of highly confined resonances and hotspots while simultaneously providing the opportunity to use AFM repositioning. The field of nanophotonics includes a diverse library of materials for the fabrication of resonators, and here we demonstrate additional functionalities and applications enabled by adding thin crystals of TMDs to the list.

## Methods

### Sample Fabrication

#### WS_2_ Exfoliation

WS_2_ flakes were mechanically exfoliated from bulk crystal (HQ-graphene) onto a nominally 290 nm SiO_2_ on silicon substrate. Large flakes with recognizable crystal axes via straight edged sides at 120° to each other were identified, and their positions within the sample were recorded for further patterning.

#### Electron Beam Lithography

Samples were spin-coated with ARP-9 resist (AllResist GmbH) at 3500 rpm for 60 s and baked at 180° for 5 min, yielding a film of 200 nm thickness. Electron beam lithography was performed in a Raith GmbH Voyager system operating at 50 kV using a beam current of 560 pA.

#### Reactive Ion Etching

Anisotropic etching to imprint the resist pattern into the WS_2_ flakes physically was carried out using a mixture of CHF_3_ (14.5 sccm) and SF_6_ (12.5 sccm) at a DC bias of 180 V and a pressure of 0.039 mbar for 40 s. Isotropic etching was achieved by using a more chemical recipe with solely SF_6_ (20 sccm) at a DC bias of 50 V and a pressure of 0.13 mbar for 40 s. Removal of the remaining resist after etching was accomplished by a bath in warm 1165 resist remover (1 h) followed by acetone (5 min) and IPA (5 min). If resist is still found on the sample, final cleaning is done in a bath of acetone (1 h) and IPA (5 min) followed by 1 h in a UV ozone treatment. In some cases, the structures were slightly overetched leading to nanoantennas with a small pedestal of SiO_2_ (<20 nm). This, however, did not lead to any noticeable changes in the photonic resonances nor in the ability to reposition the structures with AFM.

#### WSe_2_ Transfer

WSe_2_ monolayers were mechanically exfoliated from a bulk crystal (HQ-graphene) onto a (PDMS) stamp, which had previously been attached to a glass slide. Large monolayers were identified using PL imaging. The glass slide is rotated upside down and attached to a holder arm by means of a vacuum. The target substrate, consisting of WS_2_ nanoantennas on a SiO_2_ surface, was also held to a stage using the same vacuum. The WSe_2_ monolayer was slowly brought into contact with the target substrate through the use of a piezo-scanner stage. After the entire monolayer contacted the surface, the glass slide with PDMS was slowly moved away from the target substrate. The low speed of the peeling process makes use of the viscoelastic properties of the PDMS polymer and leaves the monolayer of WSe_2_ onto the substrate.

### Dark-Field Spectroscopy

Optical spectroscopy in a dark-field configuration was achieved using a Nikon LV150N microscope with a fiber-coupled output. Incident illumination from a tungsten halogen lamp in the microscope was guided to a circular beam block with a diameter smaller than that of the beam. The light was then reflected by a 50:50 beam splitter toward a 50× Nikon (0.8 NA) dark-field objective which illuminates the sample only at large angles to the normal. Reflected light from the sample is guided back through the same objective toward a fiber coupler. Because of the small diameter of the multimode fiber core used, only light reflected back at small angles to the normal is collected. The fiber from the microscope was subsequently coupled to a Princeton Instruments spectrometer and charge coupled device (CCD).

### Micro-photoluminescence Spectroscopy

In order to record the photoluminescence emitted from monolayer WSe_2_ at different regions of our sample, we used a home-built setup, which includes a pulsed diode laser at 638 nm. The sample was mounted into an Oxford Instruments flow cryostat, and the chamber was pumped to vacuum. The collimated excitation laser was passed through a 700 nm short-pass filter, a Glan-Thompson linear polarizer, and a half wave plate before being deflected by a 50:50 beam splitter and passing through a 100× (0.7 NA) Mitutoyo objective which focused the beam onto the sample. The emitted light is collected by the same objective, passes through the beam splitter to be guided through a 700 nm long-pass filter, and is focused onto the slit of a Princeton Instruments spectrometer (0.75 m) and CCD (data shown in Figure 2d,e and Supporting Information 5 and 7). The PL decay time studies utilized the pulsed laser excitation, and the emitted light was spectrally filtered (10 nm) using the exit slit of the spectrometer before it was fiber-coupled to an ID Quantique avalanche photodiode (id100) (data shown in Figure 2f and Supporting Information 7).

### Atomic Force Microscope Repositioning and Imaging

All repositioning was carried out using a JPK Nanowizard 3 Ultra AFM using Bruker SNL probes (cantilever C, nominal stiffness 0.24 N/m). First, nanoantennas were imaged in QI mode with a set point of 1 nN, a Z length of 400 nm, and a pixel time of 15 ms. To reposition the nanoantennas, the AFM was switched to contact mode and 200 nm scans were performed with a set point of 1 nN and a scan rate of 2 Hz on the substrate immediately adjacent to the nanoantenna. The fast scan axis was oriented along the desired direction of movement, and the scan area was progressively moved so that the scan overlapped with the nanoantenna to translate it in the desired direction in increments of 5–50 nm at a time. Periodically, the nanoantenna was reimaged in QI mode to check the relative position and orientation of the pillars. Final characterization after repositioning was performed in QI mode.

### FDTD Simulations

The finite-difference time-domain simulations were carried out using Lumerical Inc. software.

#### Scattering Simulations

Calculations of the scattering cross section shown in Figure 3 and Supporting Information 1 and 3 were carried out by defining the geometry of the WS_2_ nanoantennas onto a SiO_2_ substrate utilizing the refractive index of WS_2_ from ref (20). Illumination with a plane wave was sent normal to the surface using a TFSF source from the air side. The illumination was unpolarized; however, for Figure 3 the polarization was set parallel to the dimer axis. The scattered intensity was subsequently collected from a monitor set above the illumination plane (in the far-field) so that the dark-field spectroscopy experiments could be closely emulated.

#### Electric Field Intensity Simulations

Calculations of the near-field electric field intensity normalized to vacuum, shown in Figures 2b and 4a, were simulated using the same geometry and illumination scheme as for the scattering simulations (polarization is shown as a white double arrow in upper left corner of each panel) with a monitor recording the electric field 0.5 nm above the top surface of the nanoantennas or as a vertical cross section of the structure passing through the dimer axis as shown in the right-most panels of Figure 4a. For Supporting Information 8, the monitor was designed as a cross-sectional profile through the midpoint of the nanoantenna height. For Figures 2b and 4a this monitor was defined to encompass the entire cross section of the nanoantennas. For the simulations performed in Supporting Information 5, 6 and 11, only a single point 1 nm from the inside edge of the dimer nanoantenna within the hotspot was recorded.

#### Purcell Factor Simulations

Simulations of the Purcell factor were carried out using the same geometry as for the electric field intensity simulations. The illumination was achieved through a dipole source placed at different positions, 0.5 nm above the top surface of the nanoantenna with a polarization parallel to the dimer axis for Figure 4b. For the simulations displayed in Supporting Information 11, the position was set to 0.5 nm above the top surface of the nanoantenna and 1 nm from the inside edge within the electric field hotspot.

### Optical Trapping Force Simulations

We have used the 3D finite element method (COMSOL Multiphysics) to calculate the optical forces in the hexagonal dimer nanoantenna. The structure is illuminated with a plane wave propagating in a normal direction to the top surface of the structure with a polarization along the dimer axis. The background refractive index was set to that of water to enable access of the nanosphere to the trapping site. We have calculated the optical forces at the resonance of the nanoantenna, corresponding to the maximum energy enhancement. The value of the optical force is obtained by integrating the Maxwell stress tensor on the surface of the target nanoparticle.^41^ A fine mesh (resolution of ≤3 nm) has been employed in the dimer gap to calculate the electromagnetic distribution with a high accuracy, minimizing the error on the evaluation of the optical force. The computational domain has been set to a sufficiently large value (>2 μm) and surrounded by perfectly matched layers in order to avoid undesired reflection and scattering from the boundary.

## Supporting Information Available

Photonic resonances of monomer nanoantennas; scattering comparison of nanoantenna geometry; dark-field spectra of dimer nanoantennas for a range of radii; hybridization of monomer nanoantenna resonances in dimer nanoantennas; photoluminescence enhancement of WSe_2_ monolayer due to coupling to WS_2_ dimer nanoantenna photonic modes; absorption enhancement of monolayer WSe_2_ due to coupling with WS_2_ dimer nanoantenna resonances; polarization-dependent PL lifetimes of WSe_2_ monolayer emission on dimer nanoantennas; second harmonic generation enhancement due to coupling with a dimer anapole mode; further atomic force microscopy repositioning experiments; radius of curvature measurement and effect on electric field intensity; modulation of the electric field intensity and Purcell factor of WS_2_ dimer nanoantennas (PDF)

## Notes

The data sets generated during and/or analyzed during the current study are available from the corresponding author on reasonable request.
