# Supplementary material for: Transition Metal Dichalcogenide Dimer Nanoantennas for Tailored Light–Matter Interactions
Source: ACS Nano. 2022 Apr 6;16(4):6493–505. doi: 10.1021/acsnano.2c00802 (PMC9047003; doi:10.1021/acsnano.2c00802)
Supplement: Supplementary file 1 — nn2c00802_si_001.pdf [file nn2c00802_si_001.pdf]

Supporting Information for: Transition metal dichalcogenide dimer nanoantennas for tailored-light matter interactions

Panaiot G. Zotev[a*], Yue Wang[b*], Luca Sortino[a,c] Toby Severs Millard[a], Nic Mullin[a], Donato Conteduca[b], Mostafa Shagar[a], Armando Genco[a], Jamie K. Hobbs [a], Thomas F. Krauss[b], Alexander I. Tartakovskii[a***]

[a]*Department of Physics and Astronomy, University of Sheffield, Sheffield S3 7RH, UK*

[b]*Department of Physics, University of York, York, YO10 5DD, UK*

[c]*Chair in Hybrid Nanosystems, Nanoinstitute Munich, Faculty of Physics, Ludwig-Maximilians-Universität, München, 80539, Munich, Germany*

*p.zotev@sheffield.ac.uk **yue.wang@york.ac.uk ***a.tartakovskii@sheffield.ac.uk

Supporting Information 1: Photonic resonances of monomer nanoantennas

As displayed in Fig. S1(a)-(c), we measured dark field spectra for hexagonal monomer nanoantennas of three different heights (170 nm, 60 nm and 25 nm) over a range of radii (100 - 370 nm) and subsequently simulated the fabricated geometries which, as shown in Fig. S1(d)-(f), exhibit close agreement to the experimental data. Examples of individual experimentally measured and simulated spectra for hexagonal nanoantennas of different heights are shown in Fig. S1(g)-(i). We identified an electric dipole resonance with small contributions from higher order modes (ED, green).[1] More complex resonances were also present in the fabricated monomer nanoantennas such as the anapole and higher order anapole modes (HOAM,cyan), seen as minima in dark field spectra shown in the middle and right column of Fig. S1.[2]

It is clear from the data and simulation results for all heights in Fig. S1, as expected from Mie theory, that an increase in resonator radius leads to the scattering of longer wavelengths of light, thereby red-shifting the modes. The height of the nanoantennas also plays an important role in the resonance wavelength as shown in the comparisons of experiment to simulation (Fig. S1(g)-(i)) for a single radius at each height corresponding to the dashed white lines in the upper panels. Here it can be observed that a decrease in height (from 170 nm to 25 nm) blue-shifts the resonances in the nanoantenna despite the increase in radius (150 nm to 290 nm) when moving from Fig. S1(g) to (i).

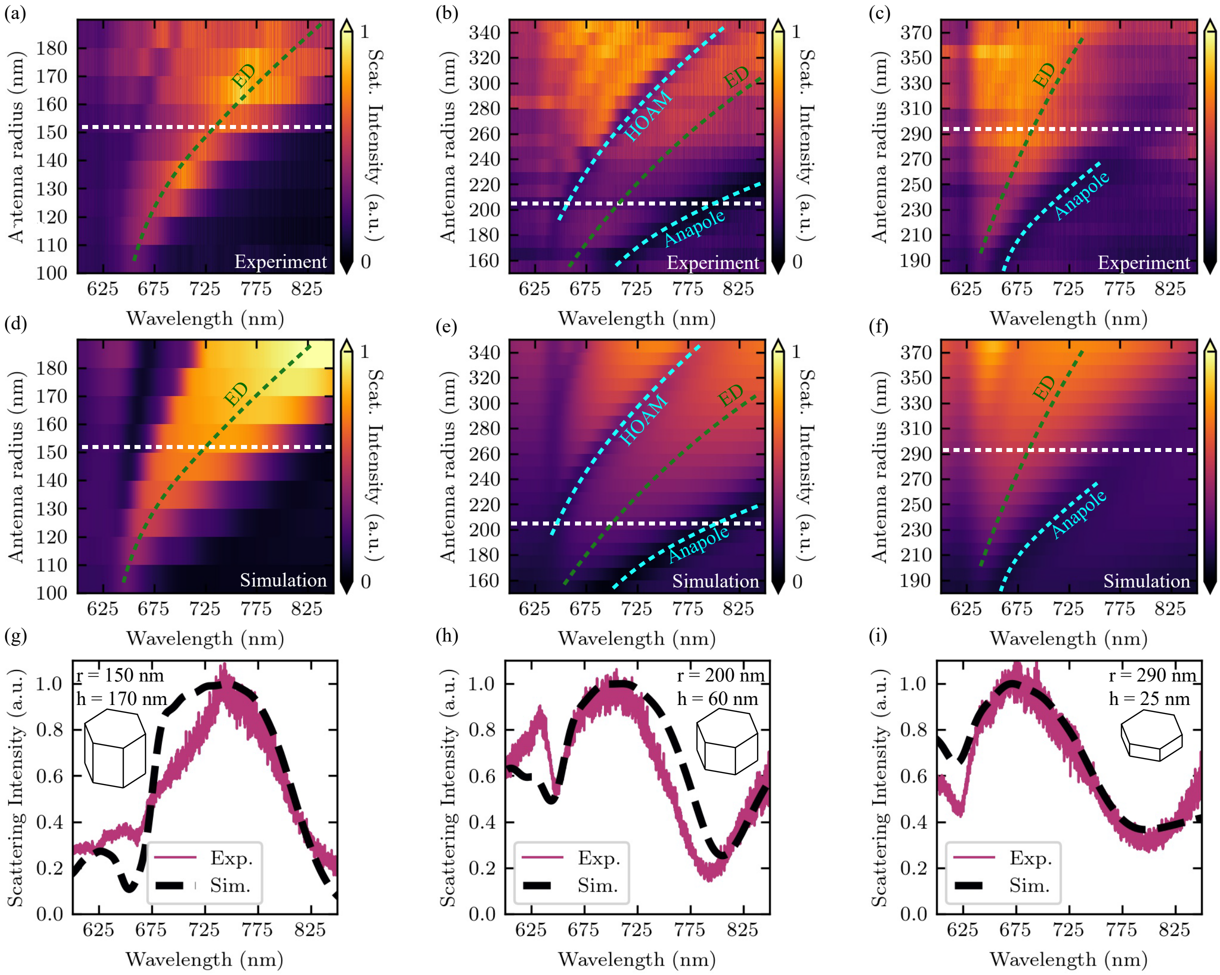

Figure S1: **Comparison of dark field spectroscopy to scattering cross section simulations for monomer nanoantennas. (a)-(c)** Dark field spectroscopy results for nanoantennas with heights of 170 nm, 60 nm and 25 nm respectively. Identified resonances include a broadened electric dipole (ED) resonance as well as anapole and higher order anapole modes (HOAM). **(d)-(f)** Scattering cross section simulations for nanoantennas with heights of 170 nm, 60 nm and 25 nm respectively. Simulations confirm the identification of ED, anapole and HOAM resonances. **(g)-(i)** A comparison between dark field spectroscopy and scattering cross section simulations for individual radii (150 nm, 200 nm, 290 nm) as defined by a horizontal dashed white line in upper panels for the three heights (170 nm, 60 nm, 25 nm).

Supporting Information 2: Scattering comparison of nanoantenna geometry

The definition of the radius leaves the possibility of a change in volume when there is a change in geometry even if the radius remains the same. Since the modes inside our structures are Mie resonances, they are heavily dependent on the geometry and the volume of the high refractive index nanoantenna.[3] As the volume increases so does the wavelength of the resonance because its fundamental mode must now fit inside a larger geometry.

In figure S2 we have compared the simulated scattering cross sections of the three different WS_2 monomer nanoantenna geometries we were able to fabricate. These calculations are done in vacuum in order to allow us to identify modes, which will broaden with the addition of a substrate. We set the same height (150 nm) and range of radii (80-250 nm) for the different geometries. We are able to identify a magnetic dipole resonance, which we denote as MD, an electric dipole resonance denoted as ED and the more complex anapole and higher order anapole mode. We also notice that the resonances blue-shift when moving from circular to hexagonal to square nanoantennas with the same radius as expected from the geometrical argument.

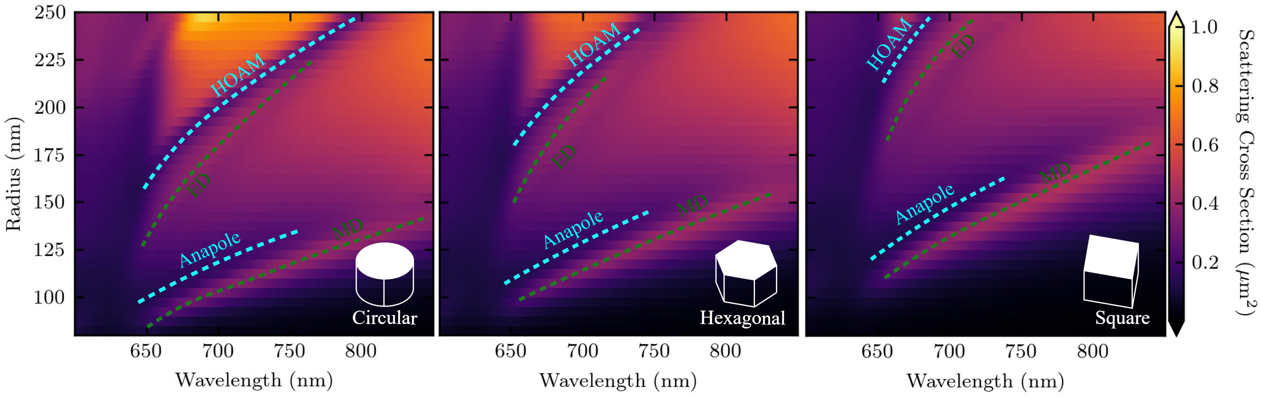

Figure S2: **Scattering cross section comparison of circular, hexagonal and square geometries.** The height of all structures was kept at 150 nm with a varying radius (80-250 nm) in order to show the blue-shift of the resonances with decreasing volume of the antenna for the hexagonal and square geometries. Identified resonances include a magnetic dipole resonance (MD), an electric dipole resonance (ED), an anapole mode and a higher order anapole mode (HOAM).

Supporting Information 3: Dark field spectra of dimer nanoantennas for a range of radii

We recorded dark field scattering spectra, under unpolarized excitation, for WS_2 dimer nanoantennas shown here in Fig. S3. The heights of the nanoantennas are all 60 nm with the radii ranging from 168 nm to 216 nm. The anapole mode, seen as the major feature of these spectra, red-shifts from 730 nm to 843 nm. This study provides evidence that the resonances of the dimer nanoantennas are just as tunable as for the monomers with a change in radius.

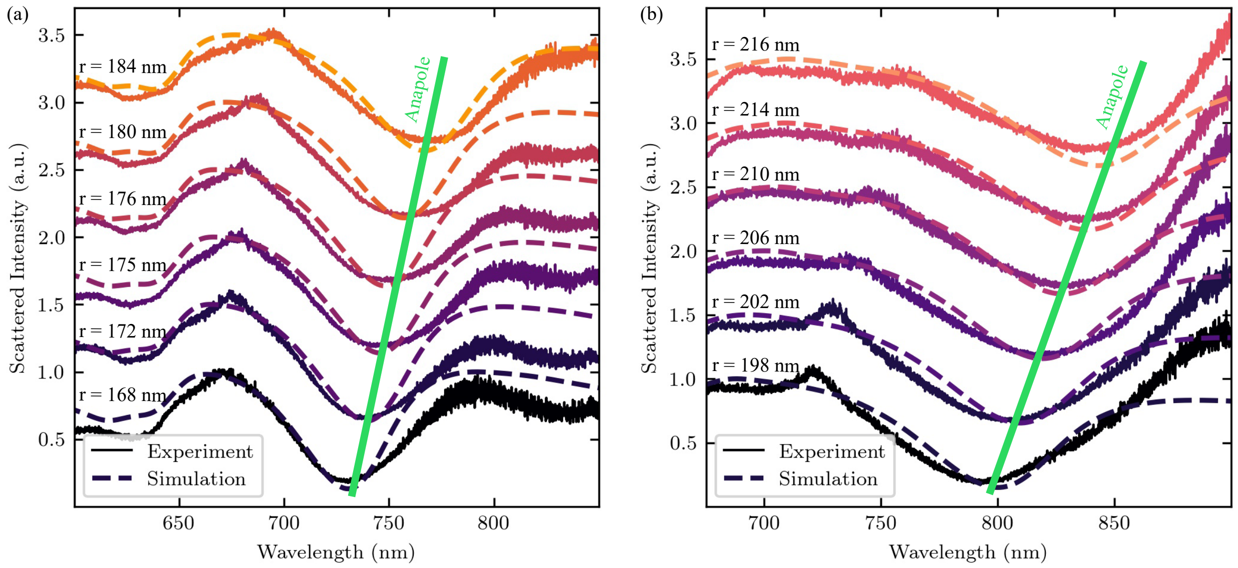

Figure S3: **Dark field scattering spectra for unpolarized excitation compared to scattering cross section simulations. (a)** Vertically offset dark field spectra compared to simulations for a range of radii (168 nm to 184 nm) with a height of 60 nm. **(b)** Same as **(a)** for a range of radii (198 nm to 216 nm) with the same height. The solid green line highlights the position of the anapole mode in these structures.

Supporting Information 4: Hybridization of monomer nanoantenna resonances in dimer nanoantennas

When two monomer nanoantennas are separated by a distance smaller or on the order of the wavelength of the individual resonances of structures, these modes begin to hybridize and therefore split energetically. This allows the formation of two new cross-polarized modes (X-pol and Y-pol) which are photonic resonances of the new dimer nanoantenna.

As shown in figure S4, we can compare the simulated scattering cross section of two nanopillars (r = 250 nm, h = 150 nm) on a SiO_2 substrate separated by a 2 μm gap to the scattering cross section of two completely isolated monomer nanoantennas. Here we simulate an excitation plane wave polarized along the dimer axis (X-pol) as shown by the yellow double arrow in the upper right corner of the figure as well as perpendicular to this (Y-pol) as shown by the red double arrow in the inset. For a separation of 2 μm, we do not observe a splitting into perpendicularly polarized modes but there is good agreement with the scattering cross section of two individual monomer nanoantennas. However, as we reduce the separation distance of the dimer to 500 nm and subsequently to 50 nm, a splitting appears at the anapole mode minimum (750-770 nm) and at the dipole resonance (670-700 nm) for the two cross polarized modes. This clearly shows the hybridization of the individual resonances of the monomer nanoantennas placed in close proximity with relation to the wavelength of the resonances.

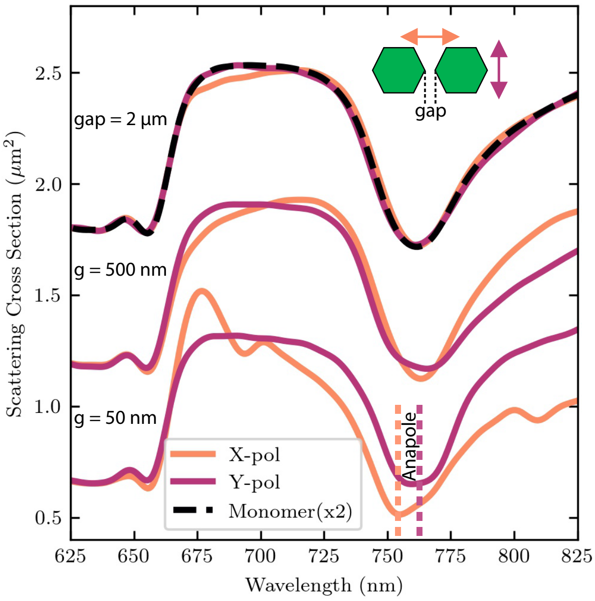

Figure S4: **Hybridization of individual resonances in dimer nanoantennas.** Simulated scattering cross sections for dimers with three different gap distances and an excitation polarization along the dimer axis (yellow) and perpendicular to it (red). At large gap distances such as 2 μm one can compare the resonances for both incident polarizations to two isolated monomer nanoantennas. At small gap distances, the modes split along the different polarization directions yielding two cross-polarized anapole and dipole resonances. Radius = 250 nm. Height = 150 nm. Spectra are vertically offset for better visualization of the results.

Supporting Information 5: Photoluminescence enhancement of WSe_2 monolayer due to coupling to WS_2 dimer nanoantenna photonic modes

In order to understand the large photoluminescence (PL) enhancement measured experimentally, we must first describe the contributing factors, which lead a photonic resonance to enhance the fluorescence of emitters

such as excitons found in a two-dimensional WSe_2 layer. The fluorescence rate enhancement (F/F_0) can be written as follows:

$$\frac{F}{F^0} = \frac{\gamma_{exc}(\lambda_{exc})}{\gamma^0_{exc}(\lambda_{exc})} \cdot \frac{q(\lambda_{em})}{q^0(\lambda_{em})} \cdot \frac{\eta(\lambda_{em})}{\eta^0(\lambda_{em})}, \quad (1)$$

where $\gamma_{exc}(\lambda_{exc})$ is the excitation rate at the wavelength of excitation, $q(\lambda_{em})$ is the quantum efficiency at the emission wavelength and $\eta(\lambda_{em})$ is the collection efficiency at the emission wavelength. All terms with ^{0} denote the same values in the absence of nanoantenna resonant effects for a monolayer on a flat SiO_2 substrate. The first term can be described as an enhancement of the rate of excitation, which is a result of the absorption of incoming light in the monolayer forming excitons, which will later recombine and lead to photoluminescence. As a reference, the number of excitons created within the monolayer at the excitation powers used in our experiments (0.02 $\mu J/cm^2$) is less than $10^{10} cm^{-2}$.[4] As the excitation rate is proportional to the electric field intensity $(|E|/|E^0|)^2$, the ratio of the rates can be estimated by simulating the electric field intensity both on the nanoantenna as well as on the SiO_2 substrate. The second factor describes an enhancement of the efficiency of emission once the monolayer is excited. This is described by the number of excitons which recombine to emit light ($q^0 = \frac{\gamma_r}{\gamma_r + \gamma_{nr}}$) where γ_r is the radiative emission rate and γ_{nr} is the nonradiative emission rate which may be due to exciton dissociation and charge transfer or auger recombination among others. When a Purcell factor is applied to this quantum efficiency, it becomes $q = \frac{F_P \gamma_r}{F_P \gamma_r + \gamma_{nr}}$. As the quantum efficiency of monolayer WSe_2 ranges from 0.06% to 5%[5–7] and therefore $\gamma_{nr} >> \gamma_r$, the enhancement of q/q^0 can simply be approximated as F_P. The last factor represents an enhancement of the percentage of light which is emitted in the direction of the collection optics ($\eta(\lambda_{em})$). The nanoantenna structures are designed to emit a larger portion of light towards the collection optics, however, their high refractive index will lead to emission towards the substrate, therefore the overall change in collection efficiency for monolayer emission from the nanoantenna region is negligible when compared to that on flat SiO_2. We have simulated the three factors contributing to the WSe_2 monolayer fluorescence on flat SiO_2 and on dimer nanoantennas with a height of 135 nm, a gap of 150 nm and range of radii (r = 100 - 250 nm) which includes those measured for the three exemplary antennas shown in Fig. 2 of the main text.

The maximum excitation rate enhancement, dependent on the electric field intensity, is displayed in Fig. S5(a) for a wavelength of 638 nm, which is identical to that of the pulsed laser used for the PL experiments. The excitation rate is highest for a dimer nanoantenna with a radius close to 200 nm. The maximum quantum efficiency enhancement, dependent on the Purcell factor (F_P), is displayed in Fig. S5(b) for an emission wavelength of 765 nm, where the peak of the red-shifted PL emission from the WSe_2 monolayer on a majority of the nanoantennas was recorded. It is evident that the Purcell factor is highest for a radius close to the one measured for dimer NA_2. This suggests that the photonic modes are close to being in resonance with the monolayer emission for this size of dimer and further confirms that the quantum efficiency enhancement can be tuned with nanoantenna size. In order to illustrate the potential quantum efficiency enhancement, we can extract the intrinsic WSe_2 monolayer quantum efficiency from reference 51 (0.06%) and multiply by the range of values in Fig. S5(b) to obtain a maximally enhanced quantum efficiency range of 0.84% - 2.36% for nanoantennas with radii of 100 - 250 nm. Fig. S5(c) shows the collection efficiency enhancement that is simulated as a percentage of light emitted within the numerical aperture (0.7) of our experimental objective for a dipole emitter. All enhancements are calculated as ratios of the simulated values on the nanoantenna to those on flat SiO_2.

The maximum simulated fluorescence enhancement factor F/F^0 due to the nanoantenna resonances, which can contribute to the increased WSe_2 monolayer PL is plotted in Fig. S5(d) together with the experimental enhancement factors (red dots). The measured $\langle EF \rangle$ falls below the maximum simulated values due to the fact that monolayer emission is not only collected from the position of maximum enhancement, but also from

surrounding areas, several nanometers from the nanoantenna edges, which will not yield such high photonic enhancement. Another contributing factor to the low measured values is due to the fact that the monolayer may not conform to each dimer nanoantenna similarly and therefore may not couple to some nanoantenna hotspots as well as others. This is due to the monolayer transfer procedure, which is not controllable enough to guarantee a uniform coupling for all nanoantennas. What can be observed, however, is that the expected enhancement factor, mostly due to the excitation rate and quantum efficiency enhancements, is expected to yield higher PL emission for a radius close to that of NA_2 as opposed to NA_1 or NA_3. This suggests that the coupling of the WSe_2 monolayer PL with the photonic resonance can be modulated by changing the nanoantenna size.

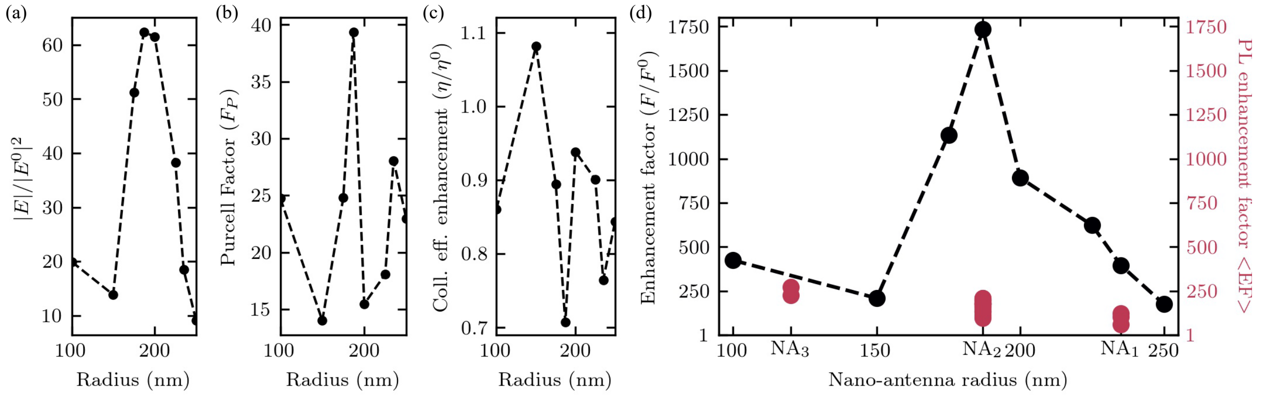

Figure S5: **Fluorescence enhancement simulations compared to experimental enhancement factors.** **(a)** Simulation of the maximum electric field intensity hotspot 0.5 nm from the top inner vertex of the hexagonal dimer nanoantennas with a height of 135 nm, a gap of 150 nm and a range of radii (100-250 nm) compared to a position 0.5 nm from flat SiO_2. Wavelength = 638 nm. **(b)** Simulation of the maximum Purcell enhancement factor for dipole emitter placed 0.5 nm from the top inner vertex of the dimer nanoantennas with the same geometry compared to a dipole placed 0.5 nm from a flat SiO_2 substrate. Wavelength = 765 nm. **(c)** Simulation of the collection efficiency enhancement for a dipole placed in the same positions as for the Purcell factor simulations. Percentage of light emitted within a cone defined by a numerical aperture (0.7) is compared to the same value simulated for a dipole 0.5 nm from a flat SiO_2 substrate. Wavelength = 765 nm. **(d)** Calculation of the total fluorescence enhancement factor F/F^0 according to equation 1 using the values simulated in **(a)**-**(c)** compared to experimental enhancement factors obtained from monolayer WSe_2 PL at nanoantenna sites.

Supporting Information 6: Absorption enhancement of monolayer WSe_2 due to coupling with WS_2 dimer nanoantenna resonances.

As evidence of excitation rate enhancement, we measure the absorption in the WSe_2 monolayer using a micro reflectance contrast setup with a Thorlabs tungsten-halogen white light source and a Princeton Instruments spectrometer (0.5 meter) and CCD. The measured positions are marked with a black and yellow circle in Fig. S6(a). The corresponding spectra on the flat SiO_2 substrate (black line) as well as at the position of nanoantenna NA_4 (yellow line) are plotted in Fig. S6(b). The monolayer absorption spectra yields a peak at 757 nm which coincides with the A exciton peak in WSe_2. The measured peak position in our experiments is red-shifted due to strain present in the monolayer as a result of cooling to liquid helium temperatures and subsequent warming.

We note that the absorption spectrum at the position of the nanoantenna yields more absorption peaks than are expected for monolayer WSe_2. In order to understand this behavior, we compare the absorption spectrum to that of the maximum electric field enhancement resulting at the top surface of a WS_2 dimer nanoantenna with a similar geometry to the one measured in experiment (r = 135 nm, h = 135 nm, gap = 150 nm). This is plotted as a red line in Fig. S6(b). The close agreement between absorption and electric field intensity peak positions provides evidence that the WSe_2 monolayer absorption is enhanced due to the photonic resonances of the underlying WS_2 dimer nanoantenna.

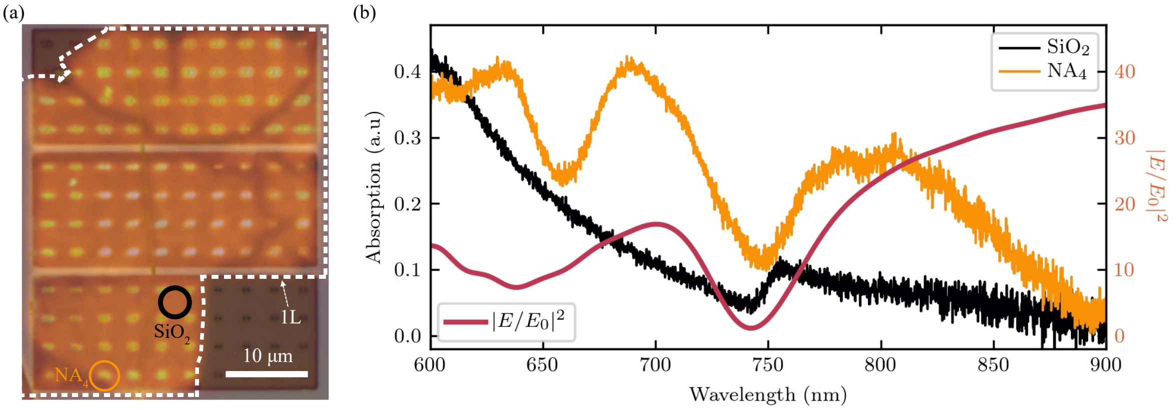

Figure S6: **WSe_2 monolayer absorption enhancement comparison to WS_2 nanoantenna electric field intensity. (a)** Photoluminescence microscope image overlaid on a bright field microscope image showing a map of the studied sample including the monolayer region. Positions at which the WSe_2 monolayer absorption was recorded are highlighted with a black (flat SiO_2) and yellow (NA_4) circle. **(b)** Absoprtion spectra of the WSe_2 monolayer measured at a position of flat SiO_2 substrate (black curve) and at the WS_2 dimer nanoantenna NA_4 (yellow curve). A simulated spectrum of the electric field intensity enhancement within the inner hotspot of a WS_2 dimer nanoantenna with a geometry similar to that of the fabricated NA_4 (r = 135 nm, h = 135 nm, gap = 150 nm) is also plotted in red.

Supporting Information 7: Polarization dependent PL lifetimes of WSe_2 monolayer emission on dimer nanoantennas

Monolayer WSe_2 room temperature PL emission was recorded over a range of dimer nanoantennas (11) for two perpendicular excitation polarizations corresponding to the X-pol and Y-pol modes. Fig. S7(a) displays the PL spectra recorded for an exemplary nanoantenna site with a height of 135 nm, a gap of 150 nm and a radius of 190 nm. We observed a higher emission intensity for the X-pol mode as expected from the higher electric field intensities for this polarization. We subsequently measured the emission lifetime of the WSe_2 monolayer PL in a 10 nm wavelength window centered at the peak (gray region in Fig. S7(a)) for both excitation polarizations, shown in Fig. S7(b). We discovered a small decay time shortening for the X-pol mode compared to the Y-pol mode, which was repeated for the majority of the measured dimer nanoantenna sites. This also suggests that Purcell enhancement is present in the emission of most of the measured structures and can be modulated by a rotation of the excitation polarization, coupling the PL emission to either the X-pol or Y-pol mode.

Subsequently, we integrated the PL intensity within the 10 nm wavelength shown as a gray region in Fig. S7(a) and calculated an increase of 5% in the PL intensity. The shortening of the lifetime seen in Fig. S7(b) was calculated to be 2.5%. This correlates with the simulations of the fluorescence enhancement factor shown in Fig. S5, where Purcell enhancement contributed to approximately half of F/F^0.

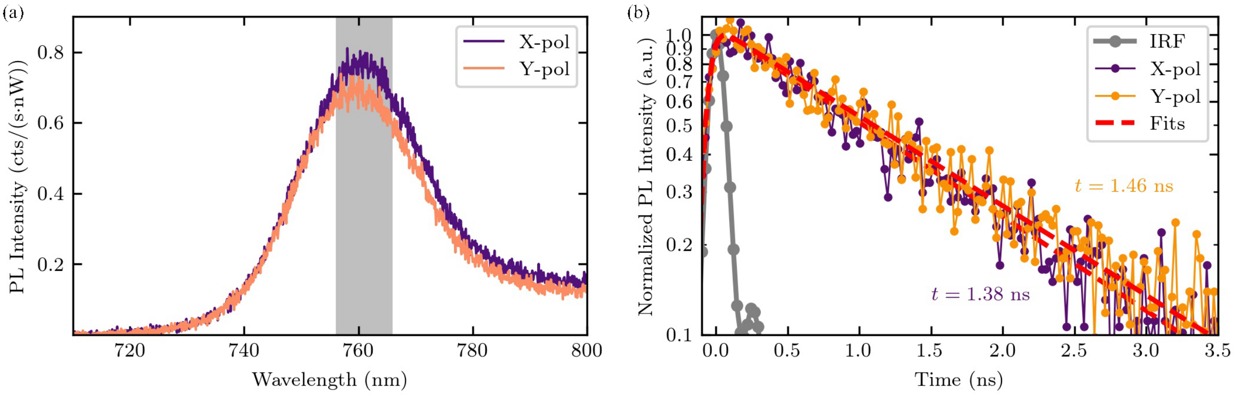

Figure S7: **Polarization dependent PL intensity and decay.** **(a)** Room temperature monolayer WSe_2 PL spectra for an excitation (638 nm) polarization aligned parallel to the dimer axis (X-pol) and perpendicular to it (Y-pol) for a nanoantenna with a height of 135 nm, a gap of 150 nm and a radius of 190 nm. The PL intensity for the X-pol mode within the wavelength range in the gray area is 5% higher than for the Y-pol mode. **(b)** PL decay traces for the X-pol and Y-pol modes on the same dimer nanoantenna as shown in **(a)**. Red dashed lines are fits of the traces, which yield decay lifetimes of 1.38 ns and 1.46 ns. The X-pol mode coupled emission yields a 2.5% shorter decay time than that of the Y-pol mode. The gray trace represents the instrument response function.

Supporting Information 8: Second harmonic generation enhancement due to coupling with a dimer anapole mode

Dielectric nanoantenna anapole modes have previously been used for second harmonic generation enhancement.[8–10] This is done by coupling an excitation beam to an anapole mode. As the anapole mode increases the electric field intensity inside the material of the nanoantenna, this leads to a stronger SHG signal. In previous studies involving TMDs, this has been accomplished using the anapole mode of a circular monomer nanoantenna.[9,10] As seen from Supporting Information 1, the fabricated hexagonal WS_2 monomer nanoantennas also host anapole modes. Moreover, WS_2 hexagonal dimer nanoantennas have also been shown to host anapole resonances in Supporting Information 3. Similar to dipole or quadrupole resonances, the anapole modes in monomers will hybridize when a dimer nanoantenna is formed. This hybridization will lead to confinement of a portion of the electric field intensity outside of the nanoantenna geometry. Figs. S8(a) and (b) show the induced electric field intensity of the anapole mode in and surrounding a monomer (r = 200, h = 60 nm) and dimer (r = 200 nm, h = 60 nm, g = 70 nm) nanoantenna respectively at the midpoint of their height (30 nm) for an excitation polarization perpendicular to the dimer axis. However, it is interesting to note that electric field intensity antinodes in the dimer nanoantenna at this small gap separation are higher than those for the monomer signifying more tightly confined light.

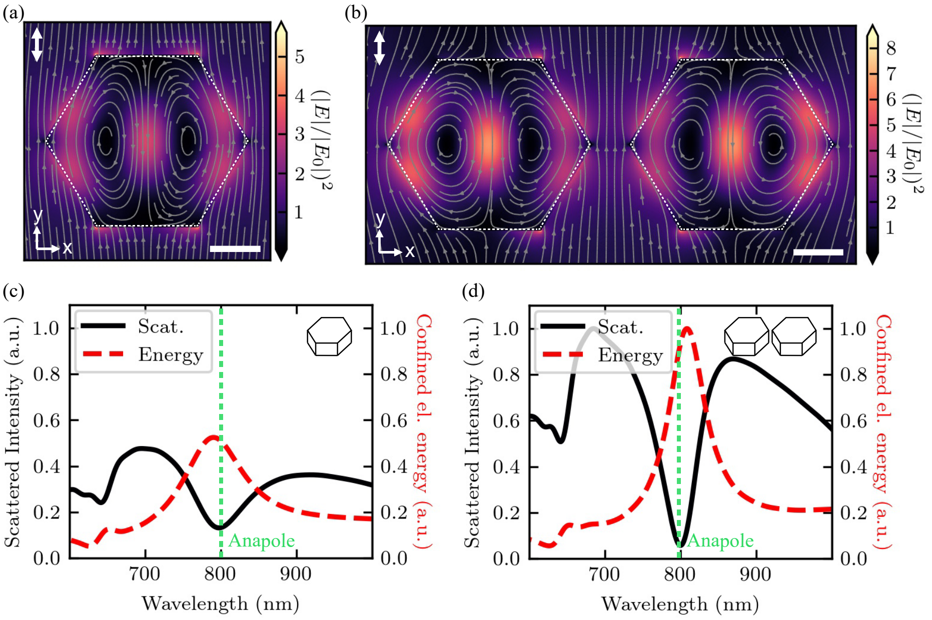

Figure S8: **Comparison of near-field electric field intensities and confined electric energies for the monomer and dimer anapole modes.** **(a),(b)** Electric field intensity spatial distributions for a cross-section at mid-height (30 nm) for a monomer (r = 200 nm, h = 60 nm) and dimer (r = 200 nm, h = 60 nm, g = 70 nm) nanoantenna respectively at the anapole mode wavelength. The double arrows in the upper left corner of both **(a)** and **(b)** indicate the polarization direction of the excitation. The grey lines represent the electric field in and surrounding the nanoantenna at this cross section. Dashed white outlines represent the physical edges of the structure. Scale bars = 100 nm. **(c),(d)** Simulated scattering cross section and confined electric energy inside the mid-height cross section of the monomer and dimer nanoantennas respectively. The scattering and electric energy spectra are normalized to the maximum of the dimer response for comparison. The vertical dashed, green line indicates the position of the anapole mode.

Second harmonic generation is a second order process, which, to a dipole approximation, is a surface phenomenon, which scales with the electric energy at the excitation wavelength squared, $|W_E^{(S)}|^2$.[8] Therefore, we calculated the electric energy confined to the inside of the nanopillars at the surface described for Figs. S8(a) and (b) using the following definition:[8]

$$W_E^{(S)} = n^2/2 \int\int \|E(r, \lambda_A)\|^2 dS, \tag{2}$$

where n is the refractive index and $\|E(r,\lambda)\|^2$ is the electric field intensity. The integration was carried out over the inside surface of the nanoantennas for a range of wavelengths. Figs. S8(c) and (d) display the confined electric energy (red dashed curves) inside the monomer and the dimer nanoantenna respectively with a simulated scattering cross section (black solid curves) for each as a reference to the anapole mode. The scattering cross sections show a deeper minimum for the dimer anapole mode and an increased maximum confined electric energy (by a factor of 1.9) for the dimer nanoantenna when compared to the monomer. Two uncoupled monomers would yield a slightly higher electric energy than a dimer nanoantenna leading to higher SHG enhancement in the monomer when the electric energy is squared.[8]

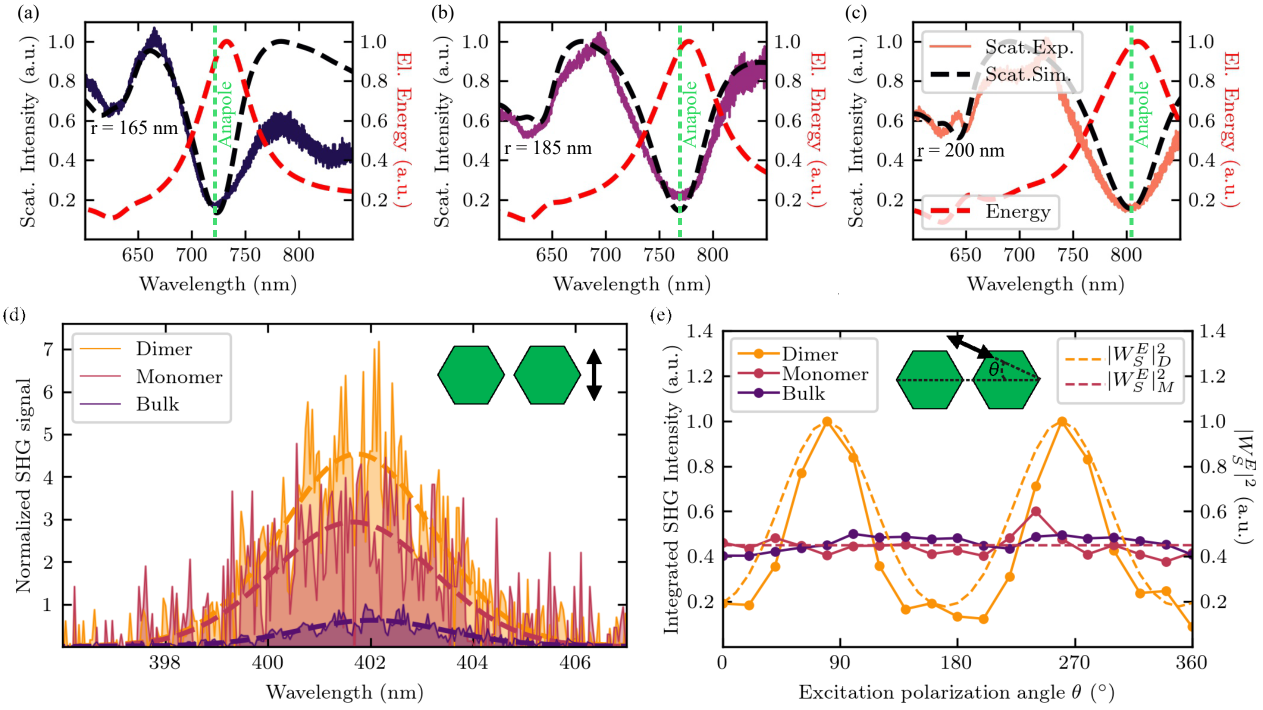

Figure S9: **Second harmonic generation enhancement of hexagonal dimer nanoantennas through coupling to an anapole mode.** **(a)-(c)** Dark field spectroscopy experiments (blue, purple, yellow curves) compared to scattering cross section simulations (black dashed curves) of dimer nanoantennas with different radii (r = 165 nm, r = 185 nm , r = 200 nm), a gap of 130 nm and a height of 60 nm. Dashed red curves correspond to simulated electric energy confined in the nanoantenna at mid-height. The anapole mode is highlighted with a vertical green dashed line. **(d)** Spectrum of second harmonic generation normalized to the excited area from a dimer nanoantenna (yellow, r = 205 nm, h = 60 nm, g = 130 nm), a monomer nanoantenna (red, r = 215 nm, h = 50 nm) and a bulk crystal (purple, h = 60 nm). Excitation polarized perpendicular to the dimer axis as shown by the double black arrow in the inset. Dashed curves represent a Gaussian fit to the data. **(e)** Normalized, integrated polarization resolved second harmonic signal emitted from the dimer (yellow, r = 200 nm, h = 60 nm, g = 130 nm) and monomer (red, r = 240 nm, h = 50 nm) nanoantennas as well as the bulk crystal (purple, h = 60 nm). The linear polarization of the excitation was set at an angle defined with respect to the dimer axis explained by the inset. Dashed curves represent the scaled polarization dependence of $|W_S^E|^2$ simulated for dimer (yellow) and monomer (red) nanoantennas with a similar geometry.

In order to experimentally evaluate the SHG enhancement of the fabricated WS_2 hexagonal nanoantennas, we firstly identified anapole resonances in several dimers with a varying radius (r = 165 nm, r = 185 nm, r = 200 nm), a gap of 130 nm and a height of 60 nm by studying their scattering spectra. The scattering intensities of these structures, shown in Fig. S9(a)-(c) are in close agreement with FDTD simulations of the scattering cross sections for a similar geometry (black dashed curves). The presence of anapole resonances is corroborated by simulations of the electric energy inside each nanoantenna (red dashed curve) at the midpoint of its height. An indication that these resonances are anapole modes is the coincidence of a maximum in the confined electric energy and a minimum in the scattering cross section. As the anapole mode arises due to the destructive interference between an electric dipole and a magnetic toroidal resonance, it will not scatter light but instead confine it and increase the energy inside the dielectric structure.[2]

We subsequently recorded second harmonic generation spectra from a dimer (r = 205 nm, h = 60 nm, g = 130 nm) and monomer nanoantenna (r = 215 nm, h = 50 nm), which both host anapole modes near 800 nm, as well as from a bulk crystal (h = 60 nm). We used a 804 nm pulsed Spectra-Physics Ti-sapphire mode-locked fs laser excitation linearly polarized perpendicular to the dimer axis (Y-pol) with an average power of 1 mW. Fig. S9(d) displays the SHG spectra (collected via a Princeton Instruments spectrometer and CCD) normalized to the excited area. For the bulk measurement, the intensity was normalized to the area of the laser spot size ($A = \pi r^2$ for r = 700 nm). The hexagonal nanoantenna measurements were normalized to their respective areas ($A_{mono} = 3r^2\sqrt{3}/2$ for the monomer with r = 215 nm and $A_{di} = 3r^2\sqrt{3}$ for the dimer with r = 205 nm).

We extracted an SHG enhancement factor by integrating the normalized second harmonic signal at the dimer nanoantenna position (yellow in Fig. S9(d)) and dividing by the similarly integrated signal from the bulk flake (purple dots), yielding a factor of 7.2. The normalized signal from the monomer was comparable to the dimer nanoantenna, which is expected from the simulations of the electric energy stored in the anapole mode of a similar geometry shown in Fig. S8.

We further studied the polarization dependence of the nanoantennas by shifting the excitation laser to 850 nm (3 mW), rotating its linear polarization and collecting integrated second harmonic signal through the use of an avalanche photodiode (APD, ID Quantique). The laser wavelength used for these experiments was chosen due to the larger difference of confined electric energy expected for the X-pol and Y-pol modes of the dimer. The normalized polarization dependent SHG intensity was recorded for a dimer (r = 200 nm, h = 60 nm, g = 130 nm), monomer (r = 240, h = 50 nm) and bulk as shown in Fig. S9(e). Upon comparison of the measured signals, we observed a clear polarization of the second harmonic signal for the dimer nanoantenna while the same is not evident for the bulk or monomer nanoantenna measurements. As the excitation polarization is rotated, the X-pol and subsequently the Y-pol modes confine incident light differently leading to the observed polarization dependence. The electric field is mainly confined to the outside edges of the nanoantenna for the X-pol anapole mode, thereby not contributing to the SHG enhancement. For the Y-pol mode, however, the confinement is mostly inside the nanoantenna structure. The position of the confined electric field in the monomer and bulk does not change, therefore, leading to no polarization dependence of the SHG enhancement. We compared the experimental data to simulations of the relative SHG signal (calculated as the square of the electric energy as described above) with a varying linear polarization, shown in Fig. S9(e) as dashed curves, and observed good agreement for both structures.

Additionally, we studied the degree of linear polarization of the SHG enhancement that we discovered in the WS_2 dimer nanoantennas. We performed simulations of the square of the electric energy inside the nanoantenna structure at the anapole resonance $|W_E^S|^2$ for the X-pol and Y-pol modes and defined a degree of linear polarization as follows:

$$DOP_{SHG} = \frac{|W_E^S|_Y^2 - |W_E^S|_X^2}{|W_E^S|_Y^2 + |W_E^S|_X^2}, \tag{3}$$

where $|W_E^S|_X^2$ and $|W_E^S|_Y^2$ represent the square of the electric energy confined in the anapole mode for the X-pol and Y-pol modes respectively. Fig. S10(a) shows the DOP_{SHG} over a range of excitation wavelengths for a dimer nanoantenna of 60 nm height, 200 nm radius, 100 nm gap and an anapole resonance at 800 nm. The highest DOP_{SHG} for this geometry is >80%. An important observation is that degree of linear polarization is positive for some excitation wavelengths and negative for others, indicating that the mode, which will provide the highest SHG enhancement at that wavelength is the Y-pol and X-pol mode respectively. This suggests that by varying the excitation wavelength, one can induce opposite SHG enhancement polarizations in the dimer nanoantenna and therefore, modify the polarization of the SHG signal. This can be advantageous for the realization of optical logic gates.[11]

We subsequently studied the change in the degree of polarization over a range of gap sizes. Fig. S10(b) displays the excitation wavelength dependencies for gaps of 0-300 nm. As the gap increases, the degree of linear polarization of the SHG enhancement is reduced over the entire wavelength range. This is expected as the increase in gap leads to a dimer nanoantenna with less hybridized resonances and therefore a response closer to that of the monomer nanoantenna, which we have shown to exhibit no polarization dependence. Incidentally, the highest degrees of linear polarization of the SHG enhancement due to both the X-pol and Y-pol anapole resonances are observed for gaps below 50 nm, which can only be achieved through the AFM repositioning technique introduced in the main text. This highlights the importance of WS_2 dimer nanoantennas with

ultrasmall gaps for nonlinear light applications.

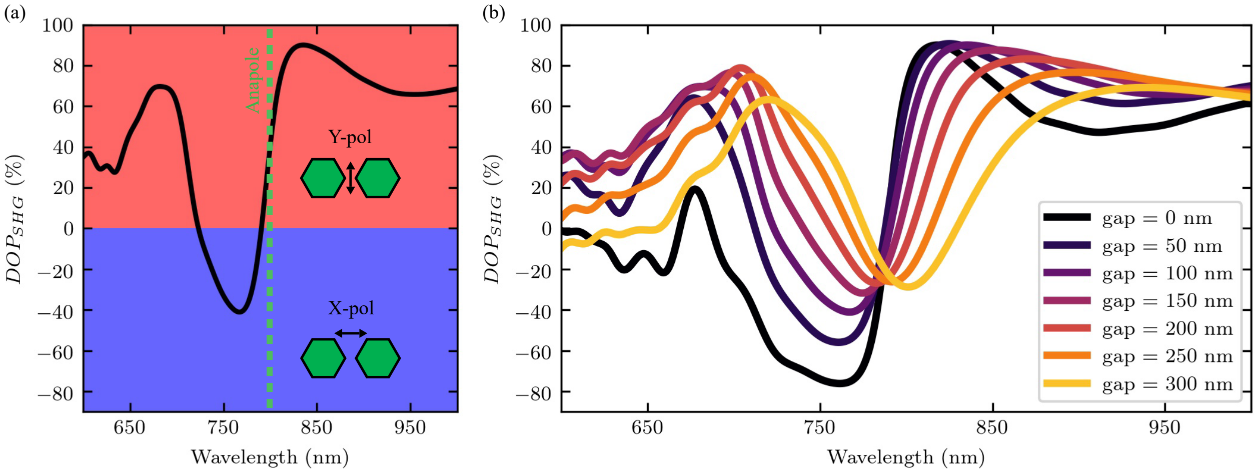

Figure S10: **Study of the degree of linear polarization of the SHG enhancement due to the anapole resonance in WS_2 dimer nanoantennas. (a)** Degree of linear polarization of the SHG enhancement over a range of excitation wavelengths for a WS_2 dimer nanoantenna with h = 60 nm, r = 200 nm, g = 100 nm. The position of the anapole resonance, at 800 nm, is shown by a green dashed line. The red (blue) region corresponds to a higher (lower) SHG enhancement in the Y-pol as compared to the X-pol mode shown by the insets. **(b)** Gap dependent degree of linear polarization of the SHG enhancement over a range of excitation wavelengths. Smaller dimer gaps lead to higher degrees of linear polarizations.

Supporting Information 9: Further Atomic force microscopy repositioning experiments

In order to provide evidence for the reproducibility of the atomic force microscope (AFM) dimer repositioning method, we show before and after scans (Fig. S11) of two more nanoantennas which have been repositioned. In Fig. S11(a) we achieve a rotation of 17° as well as a reduction of the dimer gap from 110 nm to 10 nm, which is the smallest achieved in our experiments, for a dimer with a radius of 163 nm and a height of 167 nm. In Fig. S11(b) we show a rotation of 57° and a gap reduction from 127 nm to 27 nm for a dimer with a radius of 140 nm and a height of 168 nm.

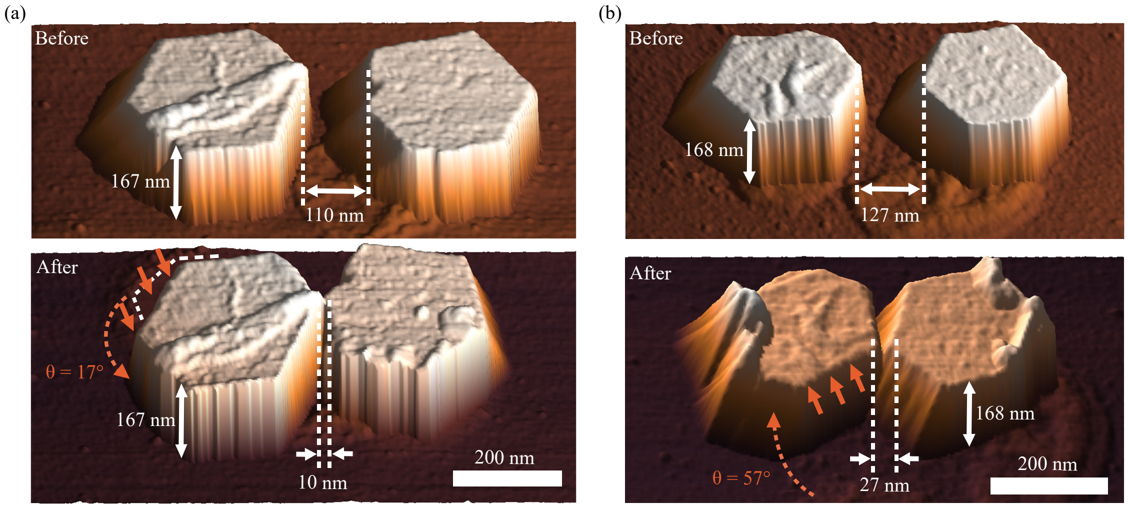

Figure S11: **Additional exemplary AFM scans before and after repositioning manipulations. (a)** AFM scans of a dimer nanoantenna before and after repositioning with a radius of 163 nm, a height of 167 nm, a reduction in gap from 110 nm to 10 nm and a rotation of 17°. **(b)** AFM scans of another dimer nanoantenna before and after repositioning with a radius of 140 nm, a height of 168 nm, a reduction in gap from 127 nm to 27 nm and a rotation of 57°.

Supporting Information 10: Radius of curvature measurement and effect on electric field intensity

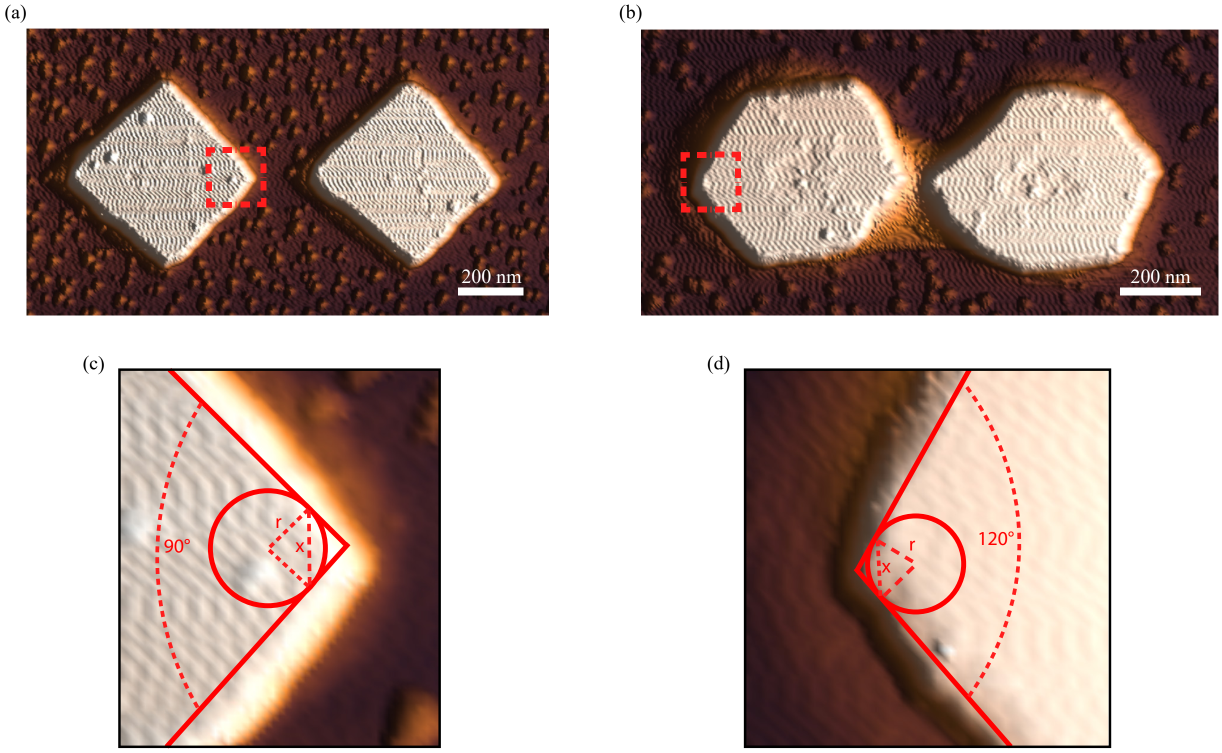

Figure S12: **AFM scans showing method of extracting vertex radius of curvature. (a),(b)** AFM scans of exemplary square (r = 265 nm, h = 84 nm, gap = 220 nm) and hexagonal (r = 240 nm, h = 135 nm, gap = 90 nm) dimer nanoantennas. **(c),(d)** Portions of the previous AFM scans shown as dashed red squares in **(a)** and **(b)**. These are overlapped with solid red lines at 90° and 120° to each other showing the top edges of the structures with an inscribed solid circle, which is used to extract the radius (r) of curvature of the vertex. Distance measured from the AFM scan is denoted by x, which is the shortest distance between the top two edges of the structure.

In order to provide simulations of the electric field and Purcell enhancement for realistic structures, we measured the radius of curvature of vertices of many of our fabricated structures with atomic force microscopy and used recorded values in further simulations including those seen in Fig. 4 of the main text and in Supporting Information 11. Since many of the square and hexagonal structures exhibited a radius of curvature below or on the order of the maximum resolution of our AFM measurements, we used a geometric approach described below to reliably measure the radius of curvature.

In Figs. S12(a) and (b), we have shown an example of the square (r = 265 nm, h = 84 nm, gap = 220 nm) and hexagonal (r = 240 nm, h = 135 nm, gap = 90 nm) dimer nanoantenna AFM scans from which we extracted values of the radius of curvature of the vertices. For both geometries, we collected data from the top surface of the nanoantennas where the bottom of the AFM tip made contact with the structure to provide the highest resolution possible. The extracted value from the AFM scans denoted as x in Figs. S12(c) and (d) is the shortest distance between two edges which are at 90° or 120° relative to each other for the square and hexagonal geometries respectively. This can be thought of as the base of a triangle formed by two radii of curvature that perpendicularly intersect the edges of the respective structure and the angle between them can easily be calculated to be 90° and 60° for the square and hexagonal geometries respectively. The radius denoted as r in the figure can then be computed using the Pythagorean theorem.

The etching mechanism used to fabricate the WS_2 nanoantennas may yield atomically sharp vertices, however, the range we measure with AFM varies between 22 and 40 nm as shown in Fig. S12. In order to understand how a nonzero vertex radius of curvature affects a WS_2 dimer hotspot, we simulate the maximum electric field intensity enhancement 1 nm away from the inside edge of the structure. Electric field intensity spectra for a radius of curvature (CR) ranging from 0 to 40 nm are shown in Fig. S13(a) and (b) for hexagonal and square geometries respectively. The increased volume of the structure associated with the increase in vertex radius of curvature leads to a small red-shift of the resonances. A small reduction in the maximum electric field intensity is also observed similar to results from a previous report,[12] yet this is limited to no more than 20% for both structures still inducing electric field intensity enhancement factors of $>10^3$.

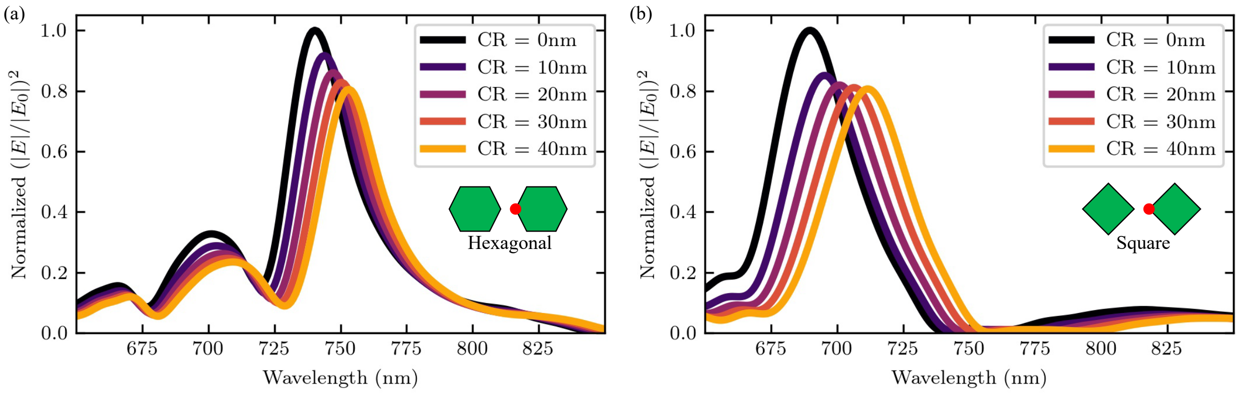

Figure S13: **Simulations of the electric field intensity in hexagonal and square WS_2 dimer nanoantenna hotspots for an increasing vertex radius of curvature.** **(a)** Simulated electric field intensity spectrum for different curvature radii (CR) at a position within the dimer gap hotspot for a WS_2 hexagonal dimer nanoantenna with radius r = 240 nm, height h = 200 nm and gap g = 10 nm. **(b)** Simulated electric field intensity spectra for different curvature radii at a position within the dimer gap hotspot for a WS_2 square dimer nanoantenna with radius r = 260 nm, height h = 150 nm and gap g = 10 nm. Insets indicate simulated geometry and red circles indicate the position of the electric field hotspot.

Supporting Information 11: Modulation of the electric field intensity and Purcell factor of WS_2 dimer nanoantennas

There are several methods of modulating the electric field confinement and the local density of optical states governing the Purcell effect within the hotspots of the dimer nanoantenna resonances. One of these methods is to increase the gap separation of the dimer constituent nanopillars. This approach can either be achieved during the patterning stage of the fabrication process or, to a greater precision, using the AFM repositioning method. We numerically studied the effect of moving the two nanopillars apart from each other (schematically depicted in Fig. S14(a)) on the electric field intensity and Purcell factor at the hotspots in Fig. S14(b) and (c). We also compared these effects for the three dimer designs. Both the electric field intensity and Purcell factor decrease exponentially with separation distance. The hexagonal structure yields the highest electric field confinement at small separations, however the intensity induced by the square structure is highest for large gap values (>75 nm). The Purcell factor of the square structure, however, exceeds that of the hexagonal at separation values as low as 20 nm.

Another approach available only to the hexagonal and square nanoantennas takes into account the control of the alignment of the two nanopillars relative to each other. One method to achieve such alignment was previously shown in Fig. 3 of the main text, while another method utilizes the chemical etching mechanism, which etches faster in the armchair axis of the crystal. Therefore, two nanopillars fabricated spatially close to each other will always have the same orientation with respect to the symmetry of the original crystal. However, if the dimer nanoantenna design is rotated at the electron beam patterning stage of the fabrication procedure, as displayed in Fig.S14(d), the relative orientation of the antennas will have changed along the new axis connecting their centers, thereby, positioning their vertices closer or further apart from each other. As shown in Fig.S14(e) and (f), this can lead to a more subtle modulation of the electric field intensity and the Purcell enhancement for a quantum emitter located within the hotspots. The range of angles available to the hexagonal geometry is limited to 30°, while the square structure can be rotated to 45° before the electric field intensity and Purcell factor begin to increase once more. The large modulation of both electric field intensity and Purcell factor shown here requires a small gap separation. As the gap increases, the rotation will lead to smaller changes in the electric field intensity and Purcell factor.

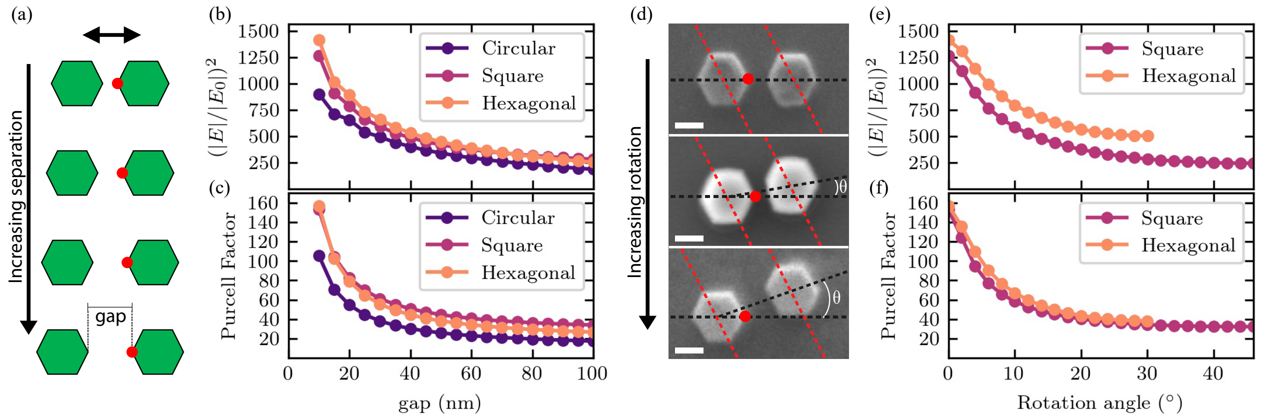

Figure S14: **Modulation of the electric field intensity and Purcell factor in dimer nanoantennas.** **(a)** Illustration of the change in dimer gap used for the simulations in **(b)** and **(c)**. **(b),(c)** Gap dependent maximum electric field intensity and Purcell factor within the hotspot of the optimized dimer designs for each geometry at wavelengths of electric field intensity. **(d)** Top-view SEM images of hexagonal dimer nanoantennas demonstrating the ability to utilize the crystal symmetry to fabricate structures with a rotation (θ) relative to the dimer axis. Red circles indicate position of electric field hotspot or dipole source for Purcell enhancement in **(e)** and **(f)**. Scale bars = 200 nm. **(e),(f)** Rotation angle dependent maximum electric field intensity and Purcell factor within the hotspot of the optimized dimer designs for the square and hexagonal geometries at the previously used wavelengths.

References

[1] Tatsuki Hinamoto and Minoru Fujii. MENP: an Open-Source MATLAB Implementation of Multipole Expansion for Applications in Nanophotonics. *Osa Continuum*, 4:1640–1648, 2021.

[2] Andrey E. Miroshnichenko, Andrey B. Evlyukhin, Ye Feng Yu, Reuben M. Bakker, Arkadi Chipouline, Arseniy I. Kuznetsov, Boris Luk'yanchuk, Boris N. Chichkov, and Yuri S. Kivshar. Nonradiating Anapole Modes in Dielectric Nanoparticles. *Nature Communications*, 6:8069, 2015.

[3] Gustav Mie. Beiträge zur Optik Trüber Medien, Speziell Kolloidaler Metallösungen. *Annalen der Physik*, 3:377–445, 1908.

[4] Shinichiro Mouri, Yuhei Miyauchi, Minglin Toh, Weijie Zhao, Goki Eda, and Kazunari Matsuda. Nonlinear Photoluminescence in Atomically Thin Layered WSe_2 Arising from Diffusion-Assisted Exciton-Exciton Annihilation. *Physical Review B - Condensed Matter and Materials Physics*, 90(15):1–5, 2014.

[5] Nur Baizura Mohamed, Feijiu Wang, Hong En Lim, Wenjin Zhang, Sandhaya Koirala, Shinichiro Mouri, Yuhei Miyauchi, and Kazunari Matsuda. Evaluation of Photoluminescence Quantum Yield of Monolayer WSe_2 Using Reference Dye of 3-Borylbithiophene Derivative. *Physica Status Solidi (B) Basic Research*, 254(2), 2017.

[6] Hyungjin Kim, Geun Ho Ahn, Joy Cho, Matin Amani, James P. Mastandrea, Catherine K. Groschner, Der Hsien Lien, Yingbo Zhao, Joel W. Ager, Mary C. Scott, Daryl C. Chrzan, and Ali Javey. Synthetic WSe_2 Monolayers with High Photoluminescence Quantum Yield. *Science Advances*, 5(1), 2019.

[7] Shrawan Roy, Anir S. Sharbirin, Yongjun Lee, Won Bin Kim, Tae Soo Kim, Kiwon Cho, Kibum Kang, Hyun Suk Jung, and Jeongyong Kim. Measurement of Quantum Yields of Monolayer TMDs Using Dye-Dispersed Pmma Thin Films. *Nanomaterials*, 10(6), 2020.

[8] Javier Cambiasso, Gustavo Grinblat, Yi Li, Aliaksandra Rakovich, Emiliano Cortés, and Stefan A. Maier. Bridging the Gap Between Dielectric Nanophotonics and the Visible Regime with Effectively Lossless Gallium Phosphide Antennas. *Nano Letters*, 17(2):1219–1225, 2017.

[9] Sebastian Busschaert, Moritz Cavigelli, Ronja Khelifa, Achint Jain, and Lukas Novotny. TMDC Resonators for Second Harmonic Signal Enhancement. *ACS Photonics*, 7(9):2482–2488, 2020.

[10] Anna Popkova, Ilya Antropov, Gleb I. Tselikov, Georgy A. Ermolaev, Igor Ozerov, Roman V. Kirtaev, Sergey M. Novikov, Evlyukhin Andrey B., Aleksey V. Arsening, Vladimir O. Bessonov, Valentyn S. Volkov, and Andrey A. Fedyanin. Nonlinear Exciton-Mie Coupling in Transition Metal Dichalcogenide Nanoresonators. *Laser and Photonics Reviews*, page 2100604, 2022.

[11] F. A. Bovino, M. Giardina, M. C. Larciprete, A. Belardini, M. Centini, C. Sibilia, M. Bertolotti, A. Passaseo, and V. Tasco. Optical Logic Functions with Nonlinear Gallium Nitride Nanoslab. *Optics Express*, 17(22):19337–19344, 2009.

[12] Hyeongrak Choi, Mikkel Heuck, and Dirk Englund. Self-Similar Nanocavity Design with Ultrasmall Mode Volume for Single-Photon Nonlinearities. *Physical Review Letters*, 118:223605, 2017.
